# Higher Circulating Vitamin D Levels Are Associated With Decreased Migraine Risk: A Mendelian Randomization Study

**DOI:** 10.3389/fnut.2022.907789

**Published:** 2022-07-08

**Authors:** Peng-Peng Niu, Xue Wang, Yu-Ming Xu

**Affiliations:** Department of Neurology, The First Affiliated Hospital of Zhengzhou University, Zhengzhou, China

**Keywords:** causal relationship, vitamin D, 25OHD, migraine, Mendelian randomization

## Abstract

**Background:**

Evidence showed the supplementation of vitamin D might have beneficial effects for migraine patients. We aimed to investigate the causal effects of serum vitamin D levels on migraine risk using two-sample Mendelian randomization (MR) method.

**Methods:**

A total of 184 independent genetic instruments for serum vitamin D levels were selected from a study in 417,580 Europeans from UK biobank. Six variants from an independent study were obtained to perform replication analysis. Summary-level data for migraine were obtained from three studies with 48,975 migraine cases, 28,852 migraine cases and 10,536 migraine cases, respectively.

**Results:**

The estimated odds ratios (ORs) per standard deviation increase in circulating vitamin D levels based on the three migraine datasets were 0.948 (95% CI = 0.883–1.016, *p* = 0.133), 0.902 (95% confidence intervals [CI] = 0.825–0.986, *p* = 0.023), and 0.880 (95% CI = 0.786–0.984, *p* = 0.025), respectively. Using pooled migraine summary data with no sample overlap, MR analysis showed per standard deviation increase in circulating vitamin D levels was significantly associated with a decreased migraine risk (OR = 0.916, 95% CI = 0.859–0.977, *p* = 0.008). Multivariable MR analyses, sensitivity analyses and replication analysis confirmed the association. MR analyses showed similar estimates for migraine with aura and migraine without aura but with wider 95% CIs. Mediation analysis showed the effect of vitamin D on migraine risk via pathway of serum calcium was corresponding to an OR of 1.003 (95% CI = 1.001–1.005) and a proportion mediated of 3.42%. The reverse MR analysis showed migraine might not affect vitamin D levels.

**Conclusion:**

This two-sample MR study showed genetically determined increased circulating vitamin D levels are associated with decreased migraine risk. The effect seems consistent across different migraine subtypes. In addition, the role of serum calcium in mediating the association between vitamin D and migraine is negligible. Future large well-designed randomized trials are warranted to assess the effects of vitamin D supplementation for migraine patients, especially in those with vitamin D deficiency.

## Introduction

Migraine is characterized by recurrent attacks of moderate to severe headache, often nausea, vomiting, photophobia, and phonophobia, lasting for 4–72 h (1). The cumulative lifetime risk of migraine is up to about 33% in women and 18% in men in the general population. The estimated 1-year prevalence in the general population is ~15% (2). As one of the leading chronic neurological disorders, migraine imposes a heavy burden on patients and society (2).

As a recurrent disorder, preventive medications that aim to reduce the frequency, duration, or severity of attacks have been recommended for patients with at least two migraine days per month (3). Traditional recommended migraine preventives such as β-blockers and anticonvulsant agents are often associated with unsatisfied effectiveness and tolerability. New drugs that inhibit the actions of calcitonin gene-related peptide seem promising in the management of episodic and chronic migraine (1, 4). However, more evidence is needed, especially for migraine patients complicated by cardiovascular disease (1, 5, 6). In addition, monoclonal antibodies directed against calcitonin gene-related peptide or its receptor are not recommend for pregnant woman and women with a wish to become pregnant in half a year because the possibility of fetal harm (4).

Given the safety concerns regarding conventional medications, complementary and alternative medicine has increasingly become popular in the management of many diseases including migraine (7–9). Vitamin D supplementation has been discussed to be one potential complementary therapy for migraine management in recent years. Epidemiological evidence showed patients with migraine had higher prevalence of vitamin D deficiency/insufficiency compared with controls (10–12). However, the causality remains uncertain. Migraine may lead to low serum vitamin D levels indirectly because sunlight, a trigger of migraine, may be avoided by some migraine patients (13). Although preliminary trials showed the supplementation of vitamin D could lead to an improvement of headache frequency and intensity for both children and adults, the sample sizes were small (14–16).

**Figure 1:**
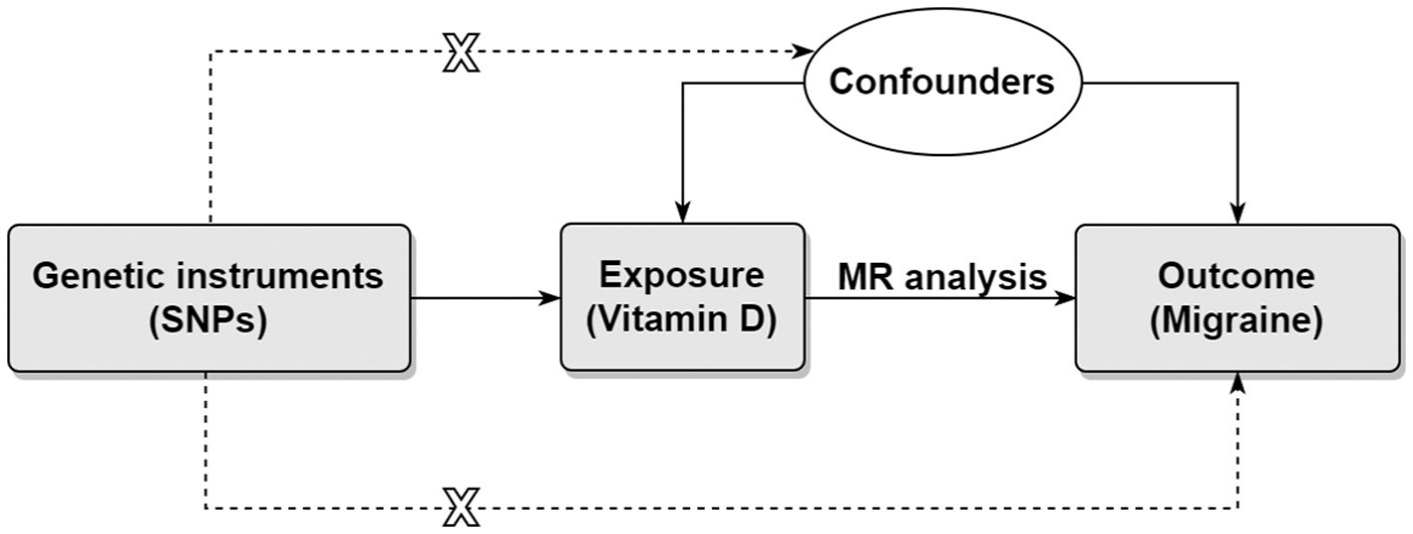
Directed acyclic graph representing the Mendelian randomization framework. There were three core assumptions for two-sample MR method. Assumption 1: the instrument SNP is associated with the exposure of interest. Assumption 2: the instrument SNP does not affect outcome via pathways other than the exposure. Assumption 3: the instrument SNP is not associated with confounders. Horizontal pleiotropy will present if the effect of the instrument SNP on the outcome was all or partially via other pathways. MR, Mendelian randomization; SNP, single nucleotide polymorphism.

Using genetic variants as instrumental variables, the Mendelian randomization (MR) method can be used to simulate randomized controlled trial because genetic variants are randomly allocated by nature (17, 18). Therefore, MR study is less prone to reverse causality bias and confounding bias compared with conventional observational study. In the present study, we aimed to assess the causal effects of genetically determined circulating vitamin D levels on risk of migraine using two-sample MR method (Figure 1).

## Materials and Methods

Informed consent and ethical approval were obtained in the original studies. We followed recent published guideline to perform the present MR study (19). Table 1 shows the datasets that we included.

**Table 1:** Characteristics of GWAS datasets.

**Trait**	**Study**	**Data source**	**Sample size**	**Ancestry**	**Migraine definition**
**Exposure**					
25OHD	Revez et al. (20)	UK biobank	417,580 individuals	European	Not applicable
25OHD	Jiang et al. (21)	31 studies	79,366 individuals	European	Not applicable
**Outcome**					
Any migraine	Hautakangas et al. (22)	21 studies from Gormley et al. (23)^†^	29,209 cases and 172,931 controls	European	Self-reported and ICHD-II
		UK biobank	10,881 cases and 330,170 controls	European	Self-reported
		GenerISK (generisk.fi)	1,084 cases and 4,857 controls	European	Self-reported
		HUNT	7,801 cases and 32,423 controls	European	Self-reported or modified ICHD-II
Any migraine	Choquet et al. (24)	UK biobank	17,532 cases and 465,435 controls	European (94.19%)	Self-reported
		GERA cohort	11,320 cases and 60,282 controls	European (81.53%)	A algorithm and ICD-8, ICD-9 and ICD-10
Any migraine	FinnGen (Release 6)	FinnGen study	10,536 cases and 208,845 controls	European	ICD-8, ICD-9 and ICD-10
MA	Gormley et al. (23)	12 studies	6,332 cases and 144,883 controls	European	Self-reported and ICHD-II
MA	FinnGen (Release 6)	FinnGen study	4,366 cases and 208,845 controls	European	ICD-8, ICD-9 and ICD-10
MO	Gormley et al. (23)	11 studies	8,348 cases and 139,622 controls	European	Self-reported and ICHD-II
MO	FinnGen (Release 6)	FinnGen study	3,924 cases and 208,845 controls	European	ICD-8, ICD-9 and ICD-10

^†^*Data from 23andme was not included*.

*25OHD, 25 hydroxyvitamin D; GERA, Genetic Epidemiology Research in Adult Health and Aging; GWAS, genome-wide association study; HUNT, an acronym for the Norwegian name: Helseundersøkelsen i Nord-Trøndelag; MA, migraine with aura; MO, migraine without aura; ICHD-II, International Classification of Headache Disorders second edition; ICD, International Classification of Disease*.

### Selection of Genetic Variants

We selected independent single nucleotide polymorphisms (SNPs) from a genome-wide association study (GWAS) of 25 hydroxyvitamin D (25OHD) concentration in 417,580 Europeans from UK biobank (20, 25). Non-fasting serum 25OHD levels were assessed using the DiaSorin Liaison XL assay, which is a direct competitive chemiluminescent immunoassay (26). Total 25OHD (including 25OHD2 and 25OHD3) was measured by the assay. Individuals with 25OHD levels out of the range of 10–375 nmol/L were excluded. The median, mean and interquartile range of 25 OHD levels were 47.9, 49.6, 33.5–63.2 nmol/L, respectively. A rank-based inverse-normal transformation was applied to the 25 OHD levels before the GWAS. A linear mixed model GWAS was performed by adjusting age at time of assessment, sex, assessment month, assessment center, supplement-intake information, genotyping batch and the first 40 ancestry principal components as covariates.

To identify independent SNPs, we downloaded the full summary data for all SNPs. We next identified SNPs associated with 25 OHD levels at a genome-wide significant level (*p* < 5 × 10^−8^). Multiallelic SNPs, palindromic SNPs, SNPs with minor allele frequency less than 0.01, and SNPs located around the human leukocyte antigen region (chr6:25–34 Mb) were further excluded. We performed a clumping procedure to obtain independent SNPs (*r*^2^ < 0.01, 10,000 kb) using the European 1,000 Genome Project Phase 3 reference panel as reference. At last, a total of 184 SNPs were included as genetic instruments (Supplementary Table 1). F statistic for each SNP was calculated using the following formula: Beta^2^/SE^2^. Beta and SE denote the estimate and standard error of effect allele on 25OHD levels. The F statistics of these SNPs range from 30 to 2,482. The proportion of variance explained by each SNP was calculated using the following formula: 2 × Beta^2^ × MAF × (1–MAF) (27). MAF denotes minor allele frequency. The proportion of variance explained by all SNPs was 4.09%.

We performed a replication analysis using six independent SNPs (*p* < 5 × 10^−8^) associated with 25OHD levels from a GWAS meta-analysis in up to 79,366 European-ancestry individuals (21). These six SNPs were also significant (*p* < 5 × 10^−8^) associated with 25 OHD levels in the GWAS based on UK biobank.

### Outcome Dataset

We obtained summary-level data for migraine from four largest studies. The first study was performed by Hautakangas et al. (22). This study combined data on 102,084 migraine cases and 771,257 controls of European ancestry from five study collections including IHGC2016 (23), 23andMe, UK biobank (25), GeneRISK (generisk.fi) and HUNT All-in Headache (28). We used the combined data on 48,975 migraine cases and 540,381 controls of European ancestry from four study collections (no 23andme) in the present study. Migraine cases were determined using self-reported data or the second edition of international Classification of Headache Disorders. Note there was sample overlap between this study and the main 25 OHD GWAS. The proportion of sample overlap was up to 57.88%. However, recent study showed two-sample MR method can be safely used for large datasets (e.g., UK biobank) with large proportion of sample overlap (29). In addition, we estimated the bias caused by sample overlap using a web tool (https://sb452.shinyapps.io/overlap/) (30). The estimated bias was negligible (~ 1%). On the other hand, the statistic of variability in instrument strength across the 184 SNPs was very high (I${}_{\text{GX}}^{2}$ of 99.08%), which further ensuring the use of MR-Egger method (29).

The second study was performed by Choquet et al. (24). The authors undertook GWAS analysis of migraine in the Genetic Epidemiology Research in Adult Health and Aging cohort and UK biobank, (31) followed by a meta-analysis combining the two results. There were 28,852 migraine cases and 525,717 controls. Most of them were of European descent (92.55%). The proportions of female were 77.98% in cases and 53.22% in controls. In the Genetic Epidemiology Research in Adult Health and Aging cohort, migraine cases were determined using a migraine probability algorithm based on migraine-specific prescriptions and codes from the 9th and 10th versions of the International Classification of Disease. In UK biobank, migraine cases were determined using self-reported data. The proportion of sample overlap between exposure dataset and this study was up to 75.30%. The estimated bias was also negligible (~1%).

The third migraine dataset was performed by the FinnGen study. We obtained summary-level data from the last release (release 6) (32). There were 10,536 migraine cases and 208,845 controls. The mean age at first migraine attack was 40.27 years. The proportion of female was 82.07%. Migraine cases determined using codes from the 8th, 9^th^, and 10th versions of the International Classification of Disease. Summary-level data on migraine with aura included 4,366 cases and 208,845 controls. Summary-level data on migraine without aura included 3,924 cases and 208,845 controls.

The fourth study was performed by Gormley et al. (23). Summary data for any migraine has been included in the study by Hautakangas et al. (22). However, in the study by Hautakangas et al. (22) analysis for migraine subtypes was only performed for SNPs significantly associated with any migraine. Summary data on migraine with aura (6,332 cases and 144,883 controls) and migraine without aura (8,348 cases and 139,622 controls) were extracted from the study by Gormley et al. (23).

Supplementary Tables 2, 3 list the effects of genetic instruments on migraine and migraine subtypes.

### Mendelian Randomization Analysis

We used the multiplicative random-effects inverse variance-weighted (IVW) method as the main MR method. The random-effects IVW method will return an unbiased estimate if the horizontal pleiotropy is balanced. Other methods including MR-Egger method, weighted median method, weighted mode method, and MR-Pleiotropy Residual Sum and Outlier (MR-PRESSO) method were used as sensitive analyses. The MR-Egger method allows all genetic variants to have pleiotropic effects (33). The MR-Egger regression intercept was used to assess if there was unbalanced horizontal pleiotropy. The weighted median method will return an unbiased estimate if more than 50% of the instrument SNPs were valid. The weighted mode method will return an unbiased estimate even if the majority of the instrument SNPs were invalid (34). The MR-PRESSO method can be used to detect horizontal pleiotropic outliers (35). If outliers were detected, an outlier corrected estimate will be reported.

In addition, we performed GWAS meta-analysis to combine SNP-migraine effects (i.e., beta and se) from different outcome datasets with no sample overlap using a fixed-effects inverse-variance approach implemented in METAL (36). The resulting pooled SNP-migraine effects were used to perform downstream MR analysis. This strategy has been used in previous two-sample MR studies with more than one outcome datasets (37, 38).

We performed steiger filtering procedure to remove potential reverse causal instruments (39). Steiger-filtered SNPs with low probability of reverse causality (Steiger *p* value < 0.01) were used to perform a sensitivity analysis. Steiger-filtered SNPs were identified based on the pooled migraine dataset with largest sample size.

Previous MR analyses showed smoking, diastolic blood pressure, insomnia and serum calcium were positively associated with migraine risk, while alcohol drinking and coffee consumption might be negatively associated with migraine risk (40–43). We performed multivariable MR analyses to adjust these factors using large public available GWASs for each of them (44–46). In addition, body mass index was also adjusted (47). For SNPs that have not been found in the select GWASs, we identified them from datasets based on UK biobank in the MRC IEU OpenGWAS database (18). For coffee consumption, we used the dataset in the MRC IEU OpenGWAS database (ID: ukb-b-5237). Pleiotropic SNPs are listed in Supplementary Table 4.

Because 25OHD may indirectly increase migraine risk via increasing serum calcium levels, we performed a two-step MR mediation analysis to assess the role of serum calcium in mediating the association between 25OHD and migraine (48). For mediation analysis, we first performed a MR analysis to assess the effect (β1) of 25OHD on serum calcium levels. We next performed a MR analysis to assess the effect (β2) of serum calcium levels on migraine risk by adjusting 25OHD. The indirect effect (i.e, mediation effect) of 25OHD via pathway of serum calcium was the product of these two estimates (i.e., β1 × β2) (48). Bootstrap method was used to estimate the 95% confidence interval (CI). The proportion mediated was calculated by dividing the indirect effects by the total effect. The total effect of 25OHD on migraine risk was the estimate from uni-variable MR IVW analysis. The pooled migraine dataset with largest sample size was used to perform the multivariable MR analysis and mediation analysis. The genetic instruments and full GWAS for serum calcium levels were obtained from a GWAS with 363,228 individuals from the UK Biobank (49).

At last, we performed a reverse two-sample MR analysis to assess the causal effect of migraine on circulating 25OHD levels. Genetic instruments for migraine were obtained from the Hautakangas et al. study (22). A total of 120 independent SNPs (*r*^2^ < 0.01, 10,000 kb) were identified (Supplementary Table 5).

We used R (version 3.6.1) to perform all of the analyses. We used the R packages of TwoSampleMR and MR-PRESSO to perform the MR analyses. Steiger filtering procedure was performed using “steiger_filtering” function in the TwoSampleMR package. For MR analyses, a *p*-value small than 0.017 (0.05/3) was considered as statistical significant. A *p*-value between 0.017 and 0.05 was considered as nominally significant.

## Results

### Any Migraine

**Figure 2:**
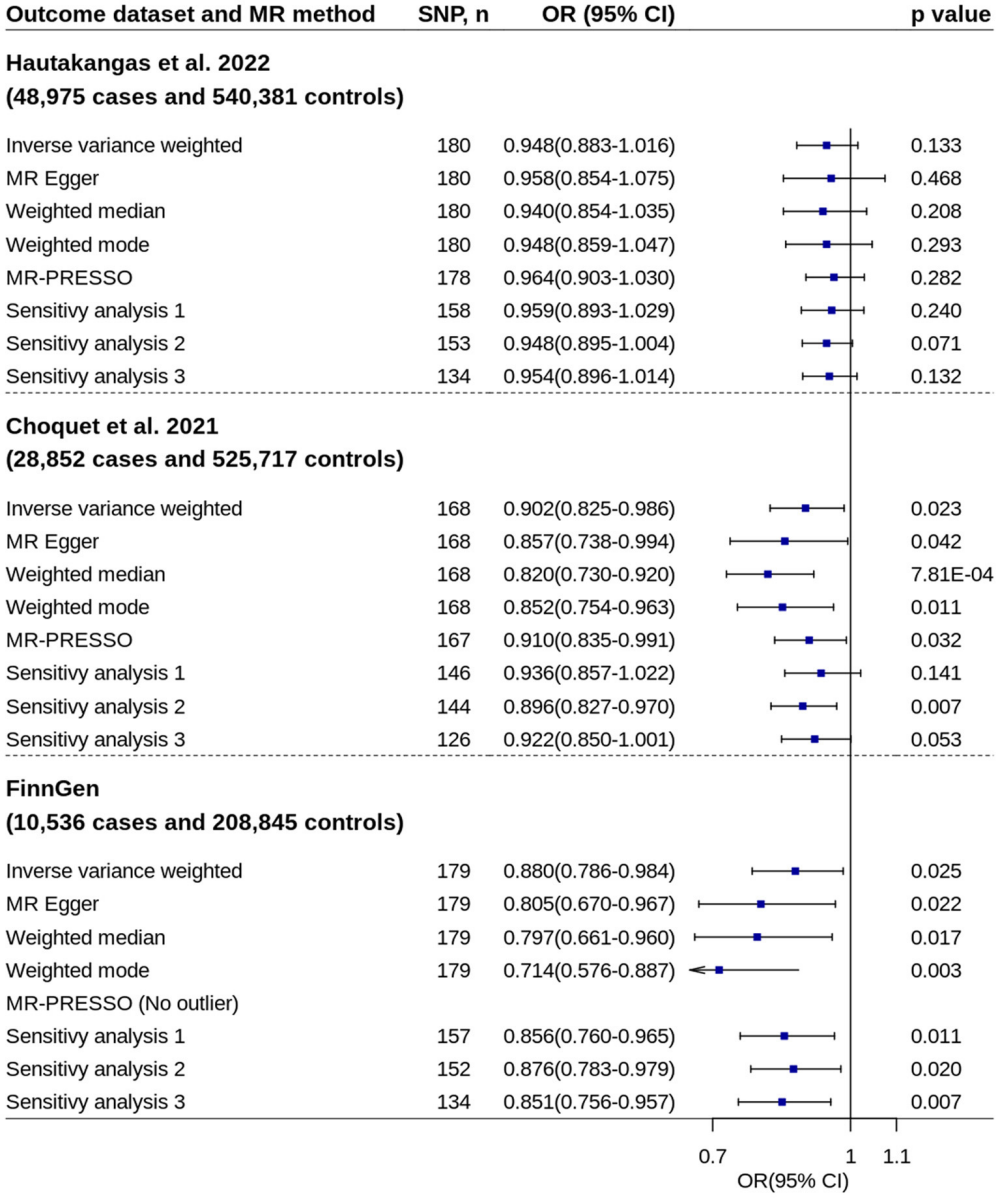
Mendelian randomization analyses of 25OHD on migraine risk. Sensitivity analysis 1: SNPs associated with potential confounders were excluded. Sensitivity analysis 2: Potential reverse causal SNPs were excluded (Steiger-filtered analysis). Sensitivity analysis 3: All SNPs excluded in sensitivity analysis 1 and sensitivity analysis 2 were excluded. 25OHD, 25-hydroxyvitamin D; CI, confidence interval; MR, Mendelian randomization; MR-PRESSO, MR-Pleiotropy Residual Sum and Outlier; OR, odds ratio; SNP, single nucleotide polymorphism.

Using migraine GWAS from Hautakangas et al. (22), two-sample MR analysis showed per standard deviation increase in circulating 25OHD levels were not associated with migraine risk (Figure 2). The odds ratio (OR) and 95% CI from IVW method were 0.948 and 0.883–1.016 (*p* = 0.133). No evidence of unbalanced horizontal pleiotropy was found by MR-Egger regression (*p* = 0.808).

Two-sample MR analyses showed increased circulating 25OHD levels were suggestively associated with decreased migraine risk by using migraine GWASs from Choquet et al. (24) and FinnGen study separately (Figure 2). The ORs based on IVW method were 0.902 (95% CI = 0.825–0.986, *p* = 0.023) and 0.880 (95% CI = 0.786–0.984, *p* = 0.025), respectively. Most of other robust MR methods and sensitivity analyses showed similar results. No evidence of unbalanced horizontal pleiotropy was found by MR-Egger regression (*p*-values: 0.397 and 0.234).

**Figure 3:**
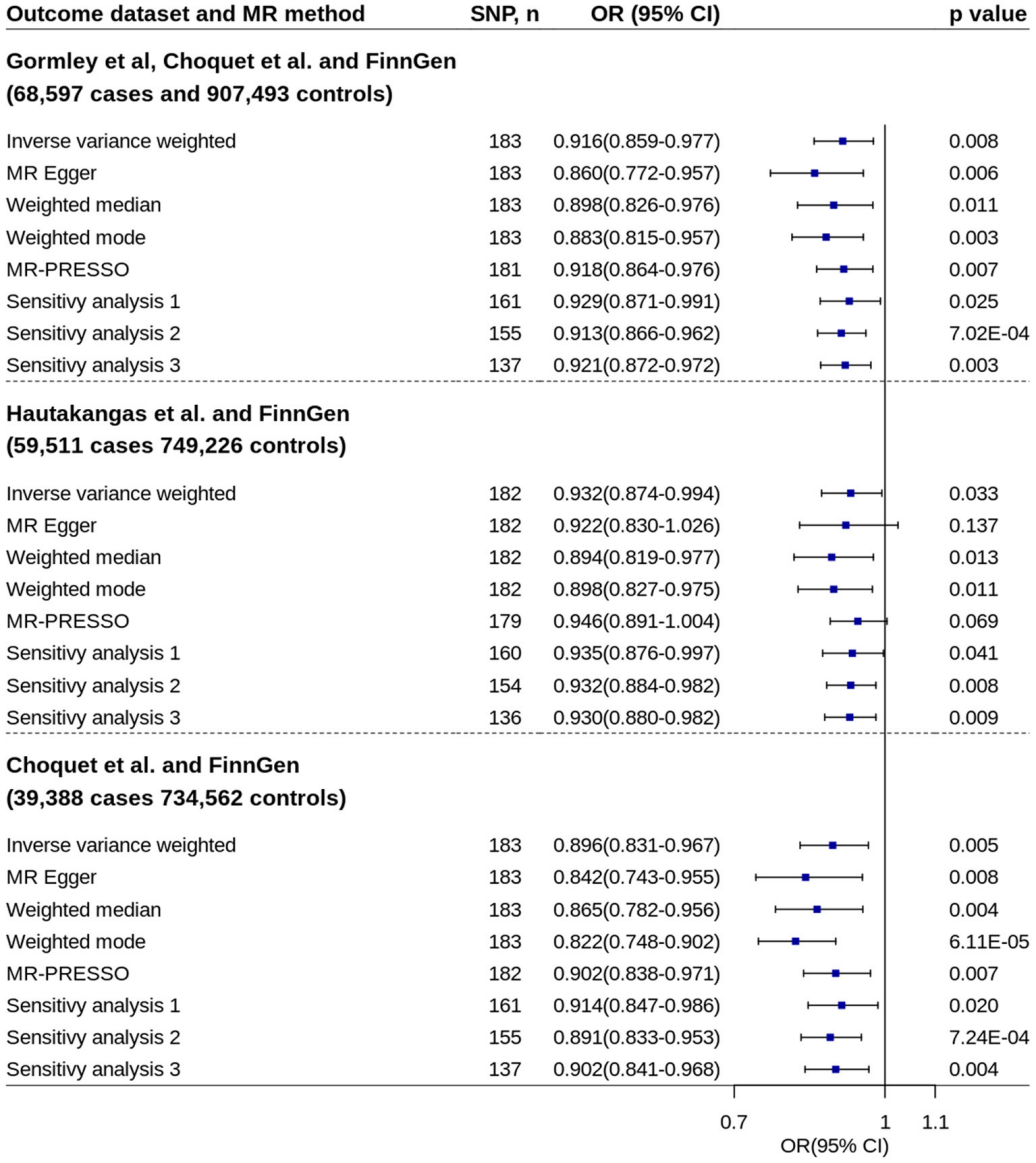
Mendelian randomization analyses of 25OHD on migraine risk using pooled migraine datasets. Sensitivity analysis 1: SNPs associated with potential confounders were excluded. Sensitivity analysis 2: Potential reverse causal SNPs were excluded (Steiger-filtered analysis). Sensitivity analysis 3: All SNPs excluded in sensitivity analysis 1 and sensitivity analysis 2 were excluded. 25OHD, 25-hydroxyvitamin D; CI, confidence interval; MR, Mendelian randomization; MR-PRESSO, MR-Pleiotropy Residual Sum and Outlier; OR, odds ratio; SNP, single nucleotide polymorphism.

Using pooled SNP-migraine effects based on samples from Gormley et al. (23), Hautakangas et al. (22) and FinnGen, MR analysis showed increased circulating 25OHD levels were associated with decreased migraine risk (IVW method: OR = 0.916, 95% CI = 0.859–0.977, *p* = 0.008) (Figure 3). Similar estimates were found by using other two pooled migraine datasets. No evidence of unbalanced horizontal pleiotropy was found by MR-Egger regression (*p*-values: 0.151, 0.812, and 0.225). The estimates remained essentially unchanged in almost all other analyses.

Supplementary Figures 1, 2 show the scatter plots of SNP-25OHD and SNP-migraine associations.

**Figure 4:**
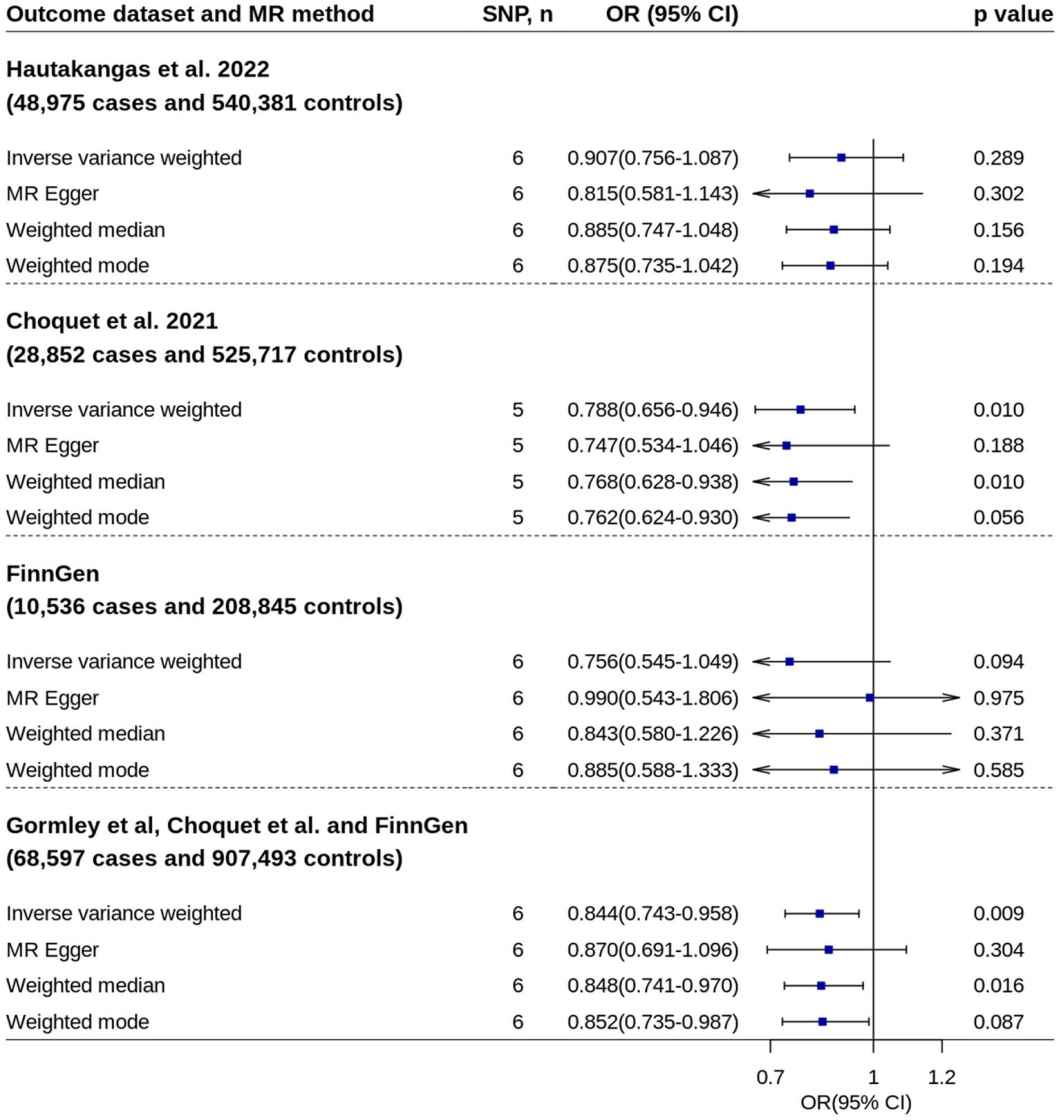
Replication analyses of 25OHD on migraine risk. No outlier was detected by MR-Pleiotropy Residual Sum and Outlier. 25OHD, 25-hydroxyvitamin D; CI, confidence interval; MR, Mendelian randomization; OR, odds ratio; SNP, single nucleotide polymorphism.

Replication analysis using six genetic instruments from an independent GWAS confirmed the association (Figure 4).

### Migraine Subtype

**Figure 5:**
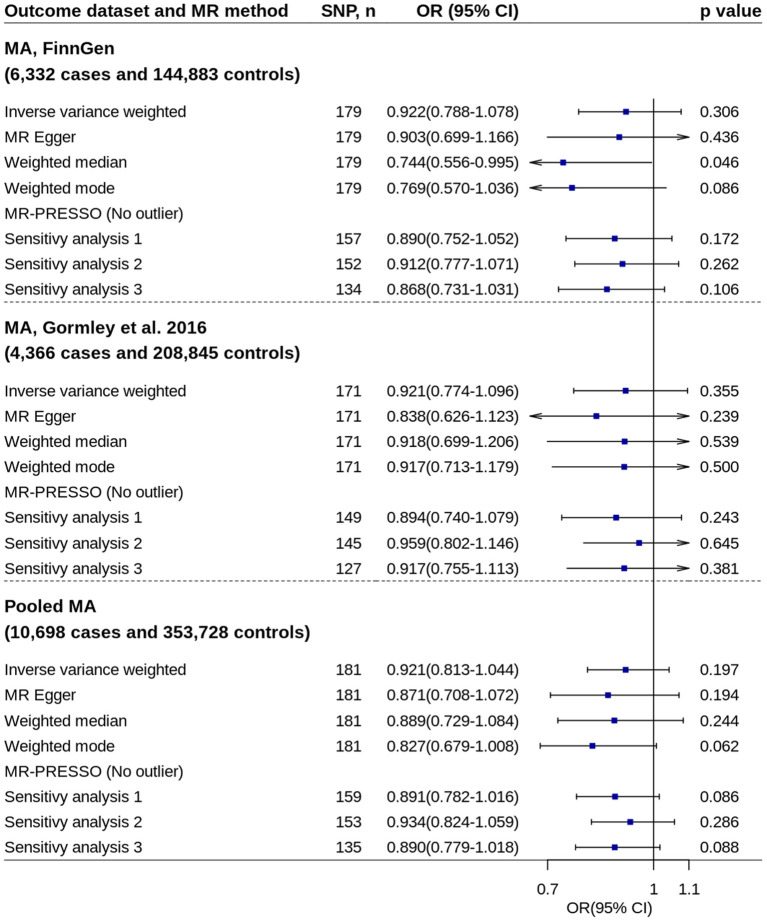
Mendelian randomization analyses of 25OHD on risk of migraine with aura. Sensitivity analysis 1: SNPs associated with potential confounders were excluded. Sensitivity analysis 2: Potential reverse causal SNPs were excluded (Steiger-filtered analysis). Sensitivity analysis 3: All SNPs excluded in sensitivity analysis 1 and sensitivity analysis 2 were excluded. 25OHD, 25-hydroxyvitamin D; CI, confidence interval; MA, migraine with aura; MR, Mendelian randomization; MR-PRESSO, MR-Pleiotropy Residual Sum and Outlier; OR, odds ratio; SNP, single nucleotide polymorphism.

**Figure 6:**
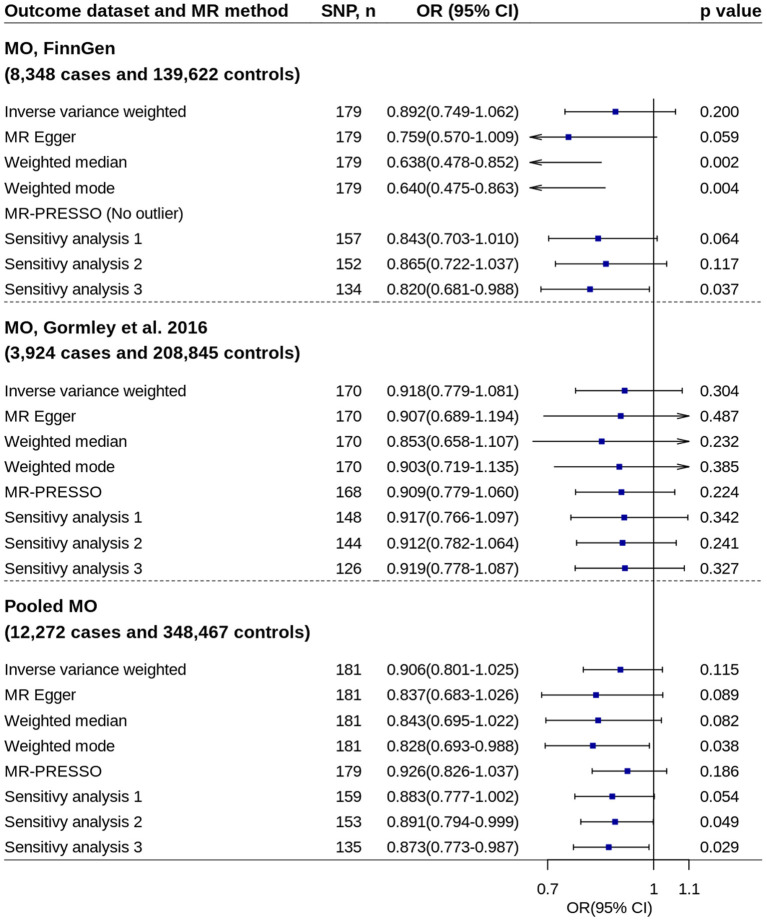
Mendelian randomization analyses of 25OHD on risk of migraine without aura. Sensitivity analysis 1: SNPs associated with potential confounders were excluded. Sensitivity analysis 2: Potential reverse causal SNPs were excluded (Steiger-filtered analysis). Sensitivity analysis 3: All SNPs excluded in sensitivity analysis 1 and sensitivity analysis 2 were excluded. 25OHD, 25-hydroxyvitamin D; CI, confidence interval; MO, migraine without aura; MR, Mendelian randomization; MR-PRESSO, MR-Pleiotropy Residual Sum and Outlier; OR, odds ratio; SNP, single nucleotide polymorphism.

All primary IVW analyses did not found significant effect of circulating 25OHD levels on risk of migraine with aura or migraine without aura (Figures 5, 6). No evidence of unbalanced horizontal pleiotropy was found by MR-Egger regression (*p*-values ≥ 0.162). However, significant or nominally significant associations were found by several robust MR methods and sensitivity analyses, especially for migraine without aura. Supplementary Figures 3, 4 show the scatter plots of SNP-25OHD and SNP-migraine associations.

Replication analysis using six genetic instruments also showed increased 25OHD levels trended toward decrease the disease risk, especially for migraine with aura (IVW method: OR = 0.779, 95% CI = 0.562–1.080, *p* = 0.134) (Supplementary Figure 5).

### Multivariable Analysis and Mediation Analysis

Multivariable MR analyses showed the benefit effect of 25OHD on risk of any migraine remained in all analyses (Supplementary Figure 6). The OR was 0.913 (95% CI = 0.855–0.975, *p* = 0.006) when adjusting all covariates at the same time. Multivariable MR analysis by adjusting serum calcium levels showed the effect of 25OHD on migraine risk increased very slightly (from an OR of 0.916 to 0.915), which suggest the role of calcium in mediating the association between 25OHD and migraine was very limited.

We further performed a two-step MR analysis to assess the role of calcium in mediating the association between 25OHD and migraine. The first MR analysis showed 25OHD was positively associated with serum calcium level (beta = 0.0504, se = 0.0194, *p* = 0.010). Using pooled migraine dataset with 68,597 migraine cases, the second MR analysis showed serum calcium levels were not significant associated with migraine risk based on IVW method (210 SNPs, OR = 1.050, *p* = 0.092). However, the effect was significant after excluding seven outlier SNPs (OR = 1.066, 95% CI = 1.013–1.123, *p* = 0.016). To avoid a conservative estimate of mediation effect, we excluded these outlier SNPs from the analysis. After excluding the seven outliers, multivariable MR IVW analysis by adjusting 25OHD showed calcium was significant associated with migraine risk (beta = 0.0601, se = 0.0267, *p* = 0.024). The two estimates (i.e., 0.0504 and 0.0601) were then multiplied together to get an indirect effect of 0.0030 (i.e., mediation effect), which corresponding to an OR of 1.003. The 95% CI of the OR was 1.001–1.005. Therefore, the indirect effect was only account for 3.42% (0.003/0.0878) of the total effect.

### Reverse Mendelian Randomization Analysis

Two-sample MR IVW analysis showed migraine was associated with decreased circulating 25OHD levels (beta = −0.016, 95% CI = −0.030 to −0.003, *p* = 0.019) (Supplementary Figure 7). However, MR-Egger regression showed evidence of unbalanced horizontal pleiotropy (*p* = 0.018). MR-Egger, weighted median and weighted mode methods all showed no association. In addition, Steiger filtered analysis showed the association attenuated to null (beta = −0.002, 95% CI = −0.016 to 0.012, *p* = 0.719), which suggest the association observed in IVW method might be attributed to reverse causality.

## Discussion

This two-sample MR study showed genetically determined increased circulating vitamin D levels are associated with decreased migraine risk. The effect seems consistent across migraine subtypes of migraine with aura and migraine without aura. In addition, the role of serum calcium in mediating the association between vitamin D and migraine is negligible.

Beside the effect on bone metabolism disorders such as rickets and osteomalacia, it has long been suggested that vitamin D has beneficial effects for many non-skeletal disorders 50]. Many observational studies showed vitamin D insufficiency was associated with extra-skeletal clinical outcomes including cardiovascular diseases, cancer, and mortality. However, except for potential benefits in cancer mortality and acute respiratory infections, randomized trials did not confirm the effects on the other outcomes including cancer, cardiovascular events, and mortality (50).

For headache disorders, as early as 1994, Thys-Jacobs reported two premenopausal women with a history of menstrually-related migraines and premenstrual syndrome (51). A combination of vitamin D and elemental calcium were prescribed to treat the premenstrual syndrome. Surprisingly, the two patients showed a significant reduction in frequency and duration of migraine headaches in 2–3 months. Later in 1996, a questionnaire study on headaches showed that the prevalence of the daily headache in the general population was higher in the northern areas of Greece than in the southern areas (52). This is consistent with the finding that vitamin D deficiency might be associated with migraine risk. A review performed by Prakash et al. confirmed the relation between the prevalence of migraine with the latitude (53). A more recently study reviewed 22 observational studies (including observational studies) and eight clinical trials that focus on vitamin D and various headache disorders. Most of the studies showed a link between serum vitamin D levels and headaches. The association was more obvious in patients with migraine than in non-migraine headaches. For example, Song et al. found that up to 77.1% (121/157) of migraine patients had vitamin D deficiency (defined as <20 ng/mL) and up to 94.9% (149/157) of migraine patients had vitamin D insufficiency (defined as <30 ng/mL) (54). In addition, monthly headache was 1.203 times more frequent in patients with vitamin D deficiency than in those without deficiency. However, no association was also observed by several other studies. Zandifar et al. showed that the prevalence of vitamin D insufficiency (<20 ng/mL) and vitamin D deficiency (<10 ng/mL) were 80 and 45.7% in cases and 81.8 and 51.8% in controls, respectively (55). Similarly, although most of preliminary clinical trials showed beneficial effect, no association was also observed. Recently, a meta-analysis including six trials with 301 migraine cases showed vitamin D supplementation was associated with decreased headache attacks per month, headache days per month, and disability grade score, but not associated with attack duration or headache severity (15).

Observational studies are often biased by confounders, as well as by reverse causality sometimes. Meta-analysis with small clinical trials is possible biased by publication bias, because small trials with positive results are easier to be published than those with non-significant results. Using two-sample MR method, our study supports the causal role of serum vitamin D in migraine.

Vitamin D receptors are expressed in almost all human cells and tissues including many regions of the brain (11). Vitamin D might has specific functions in the central nervous system by affecting cellular proliferation, acting as a neuroprotective agent and a potent antioxidant, and influencing neurotransmitters (11). Although it has been suggested that vitamin D may mainly influence migraines through its anti-inflammatory effect, randomized trials failed to confirm its effect on biomarkers of systemic inflammation (56). Vitamin D insufficiency might be a consequence of inflammatory processes involved in disease occurrence and clinical course, rather than a cause (56). There were several other possible explanations for the association between vitamin D and migraine. Vitamin D insufficiency might contribute to migraine through prompting sensitization of the second and third neurons, decreasing intestinal absorption of magnesium, and increasing the expression of nitric oxide synthase (11). However, the specific mechanisms remain to be further studied.

Although long-term vitamin D supplementation with routine dosage was considered to be safe (57), there was a concern that hypercalcemia caused by vitamin D supplementation might induce migraine attack. A previous two-sample MR study showed genetically elevated serum calcium levels and hypercalcemia were associated with increased migraine risk (43). Although the unit of serum calcium was different between the study and our study, it seems that the effect in the study was more robust compared with that of our study. However, only eight SNPs were included in the study. In addition, the result was largely attributed to one single SNP (i.e., rs1801725) that associated with hypercalcemia. The effect allele of this SNP was associated with increased risk of migraine in the GWAS (23,285 cases) that the study used (OR = 1.049, *p* = 0.0038). However, this SNP was not associated with migraine risk in the three main GWASs that we used (ORs were 1.022, 1.012 and 0.975 respectively; *p* values were 0.054, 0.354 and 0.251, respectively), nor in the pooled migraine GWAS (OR = 1.013, *p* = 0.144). Although our analysis did found that increased serum calcium levels might be associated with increased migraine risk, the effect was mild to moderate. Our mediation analysis showed the harmful effect of vitamin D supplementation for migraine via increasing serum calcium levels is negligible compared with the beneficial effect. Nevertheless, although hypercalcemia occurs rarely and usually mildly (58), it would better to monitor serum calcium levels regularly and to avoid unnecessary high vitamin D dosage to prevent hypercalcemia. In addition, some vitamin D analogs might be promising alternatives due to the low calcemic effect (59).

Our study has several strengths. First, the genetic instruments we used had enough strength. In addition, we performed replication analysis using six genetic instruments obtained from another study. Second, we used several large outcome data sources. The results were overall consistent across different outcome datasets. Third, the results are consistent across multivariable analyses, sensitivity analyses and replication analysis. Fourth, we performed MR analyses stratified by migraine subtypes. Fifth, we performed a formal mediation analysis to assess the role of serum calcium in mediating the association between 25OHD and migraine. Last, we performed a reverse MR analysis. The reverse MR analysis found migraine might not casually affect vitamin D levels.

There were several limitations of our study. First, both the exposure data and the outcome data included samples from UK biobank. Sample overlap may bias the estimates of two-sample MR study. However, recently study showed two-sample MR method can be safely used for large data from signal source such as UK biobank (29). The calculated results showed the bias due to sample overlap was negligible. In addition, the replication analysis using six genetic instruments obtained from another study with no sample overlap confirmed the association. Second, migraine is twice as common in females as in males. There were several sex-specific loci associated with migraine susceptibility in females but not in males (24). However, sex-specific MR analysis was not performed in our study because sex-specific GWAS was not available. Third, although suggestively association was found based on two separate outcome datasets, we did not found significant association based on the largest outcome dataset. This difference might be attributed to the different characteristics between the migraine samples. Fourth, the sample size for migraine with aura and migraine without aura are relatively small. However, it seems that the effect was consistent across migraine subtypes. Last, almost all of the included individuals were of European descent, which may limit the generalization of our findings for other populations.

## Conclusion

This two-sample MR study showed genetically determined increased circulating vitamin D levels are associated with decreased migraine risk. The effect seems consistent across migraine subtypes of migraine with aura and migraine without aura. In addition, the role of serum calcium in mediating the association between vitamin D and migraine is negligible. Future large well-designed randomized trials are warranted to assess the effect of vitamin D supplementation for migraine patients, especially in those with vitamin D deficiency.

## Data Availability Statement

The original contributions presented in the study are included in the article/Supplementary Material, further inquiries can be directed to the corresponding author/s.

## Ethics Statement

Ethical review and approval was not required for the study on human participants in accordance with the local legislation and institutional requirements. Written informed consent for participation was not required for this study in accordance with the national legislation and the institutional requirements.

## Author Contributions

P-PN and Y-MX contributed to conception and design of the study. XW organized the database. P-PN performed the statistical analysis. P-PN and XW wrote the first draft of the manuscript. All authors contributed to manuscript revision, read, and approved the submitted version.

## Conflict of Interest

The authors declare that the research was conducted in the absence of any commercial or financial relationships that could be construed as a potential conflict of interest.

## Publisher's Note

All claims expressed in this article are solely those of the authors and do not necessarily represent those of their affiliated organizations, or those of the publisher, the editors and the reviewers. Any product that may be evaluated in this article, or claim that may be made by its manufacturer, is not guaranteed or endorsed by the publisher.

## Abbreviations

25OHD: 25 hydroxyvitamin D
CI: confidence interval
GWAS: genome wide association study
IVW: inverse–variance weighted
MR: Mendelian randomization
MR-PRESSO: MR-Pleiotropy Residual Sum and Outlier
OR: odds ratio
SNP: single nucleotide polymorphism.
