# Supplementary material for: Higher Circulating Vitamin D Levels Are Associated With Decreased Migraine Risk: A Mendelian Randomization Study
Source: Front Nutr. 2022 Jul 8;9:907789. doi: 10.3389/fnut.2022.907789 (PMC9505695; doi:10.3389/fnut.2022.907789)
Supplement: Supplementary file 1 [file Data_Sheet_1.pdf]

Supplementary Materials

International Headache Genetics Consortium

Supplementary Table 1 Characteristics of included genetic instruments

Supplementary Table 2 SNP effects on migraine

Supplementary Table 3 SNP effects on migraine subtypes

Supplementary Table 4 Potential pleiotropic variants and their effects on confounders

Supplementary Table 5 SNPs for reverse Mendelian Randomization analysis

Supplementary Figure 1 Scatter plots of SNP-25OHD and SNP-migraine associations

Supplementary Figure 2 Scatter plots using pooled migraine datasets

Supplementary Figure 3 Scatter plots of SNP-25OHD and SNP-MA associations

Supplementary Figure 4 Scatter plots of SNP-25OHD and SNP-MO associations

Supplementary Figure 5 Replication analyses of 25OHD on risk of migraine subtypes

Supplementary Figure 6 Multivariable Mendelian randomization analyses

Supplementary Figure 7 Reverse Mendelian randomization analysis

International Headache Genetics Consortium

Verneri Anttila[1,2,3], Ville Artto[4], Andrea C Belin[5], Anna Bjornsdottir[6], Gyda Bjornsdottir[7], Dorret I Boomsma[8], Sigrid Børte[9,10,11], Mona A Chalmer[12], Daniel I Chasman[13,14], Bru Cormand[15], Ester Cuenca-Leon[16], George Davey-Smith[17], Irene de Boer[18], Martin Dichgans[19,20], Tonu Esko[21], Tobias Freilinger[22,23], Padhraig Gormley[24], Lyn R Griffiths[25], Eija Hämäläinen[26], Thomas F Hansen[12,27], Aster VE Harder[18,28], Heidi Hautakangas[26], Marjo Hiekkala[29], Maria G Hrafnsdottir[30], M. Arfan Ikram[31], Marjo-Riitta Järvelin[32,33,34,35], Risto Kajanne[26], Mikko Kallela[4], Jaakko Kaprio[26], Mari Kaunisto[29], Lisette JA Kogelman[12], Espen S Kristoffersen[36,37,38], Christian Kubisch[39], Mitja Kurki[40], Tobias Kurth[41], Lenore Launer[42], Terho Lehtimäki[43], Davor Lessel[39], Lannie Ligthart[8], Sigurdur H Magnusson[7], Rainer Malik[19], Bertram Müller-Myhsok[44], Carrie Northover[45], Dale R Nyholt[46], Jes Olesen[12], Aarno Palotie[26,47], Priit Palta[26], Linda M Pedersen[48], Nancy Pedersen[49], Matti Pirinen[26,50,51], Danielle Posthuma[52], Patricia Pozo-Rosich[53], Alice Pressman[54], Olli Raitakari[55,56,57], Caroline Ran[5], Gudrun R Sigurdardottir[6], Hreinn Stefansson[7], Kari Stefansson[7], Olafur A Sveinsson[30], Gisela M Terwindt[18], Thorgeir E Thorgeirsson[7], Arn MJM van den Maagdenberg[18,28], Cornelia van Duijn[58], Maija Wessman[26,29], Bendik S Winsvold[9,48,59], John-Anker Zwart[9,10,48]

[1]Analytical and Translational Genetics Unit, Department of Medicine, Massachusetts General Hospital and Harvard Medical School, Boston, Massachusetts, USA; [2]Program in Medical and Population Genetics, Broad Institute of MIT and Harvard, Cambridge, Massachusetts, USA; [3]Stanley Center for Psychiatric Research, Broad Institute of MIT and Harvard, Cambridge, Massachusetts, USA; [4]Department of Neurology, Helsinki University Central Hospital, Helsinki, Finland; [5]Department of Neuroscience, Karolinska Institutet, Stockholm, Sweden; [6]Neurology private practice, Laeknasetrid, Reykjavik, Iceland; [7]deCODE genetics/Amgen Inc., Reykjavik, Iceland; [8]Netherlands Twin Register, Department of Biological Psychology, Vrije Universiteit, Amsterdam, the Netherlands; [9]K.G. Jebsen Center for Genetic Epidemiology, Department of Public Health and Nursing, Faculty of Medicine and Health Sciences, Norwegian University of Science and Technology, Trondheim, Norway; [10]Institute of Clinical Medicine, Faculty of Medicine, University of Oslo, Oslo, Norway; [11]Research and Communication Unit for Musculoskeletal Health, Department of Research,

Innovation and Education, Division of Clinical Neuroscience, Oslo University Hospital, Oslo, Norway; [12]Danish Headache Center, Department of Neurology, Copenhagen University Hospital, Copenhagen, Denmark; [13]Department of Medicine, Division of Preventive Medicine, Brigham and Women's Hospital, Boston, Massachusetts, USA; [14]Harvard Medical School, Boston, Massachusetts, USA; [15]Department of Genetics, Spain Centre for Biomedical Network Research on Rare Diseases, University of Barcelona, Barcelona, Spain; [16]Pediatric Neurology Research Group, Vall d'Hebron Research Institute, Barcelona, Spain; [17]University of Bristol/Medical Research Council Integrative Epidemiology Unit, University of Bristol, Bristol, UK; [18]Department of Neurology, Leiden University Medical Centre, Leiden, the Netherlands; [19]Institute for Stroke and Dementia Research, University Hospital, LMU Munich, Munich, Germany; [20]Munich Cluster for Systems Neurology, Munich, Germany; [21]Estonian Biobank Registry, the Estonian Genome Center, University of Tartu, Tartu, Estonia; [22]Department of Neurology, Klinikum Passau, Passau, Germany; [23]Department of Neurology and Epileptology, Hertie Institute for Clinical Brain Research, University of Tuebingen, Tuebingen, Germany; [24]GSK Inc., Cambridge, Massachusetts, USA; [25]Centre for Genomics and Personalised Health, Queensland University of Technology, Brisbane, Queensland, Australia; [26]Institute for Molecular Medicine Finland, Helsinki Institute of Life Science, University of Helsinki, Helsinki, Finland; [27]Novo Nordic Foundation Center for Protein Research, Copenhagen University, Copenhagen, Denmark; [28]Department of Human Genetics, Leiden University Medical Centre, Leiden, the Netherlands; [29]Folkhälsan Research Center, Helsinki, Finland; [30]Landspitali University Hospital, Reykjavik, Iceland; [31]Department of Epidemiology, Erasmus University Medical Center, Rotterdam, the Netherlands; [32]Department of Epidemiology and Biostatistics, MRC-PHE Centre for Environment and Health, School of Public Health, Imperial College London, London, UK; [33]Center for Life Course Health Research, Faculty of Medicine, University of Oulu, Oulu, Finland; [34]Unit of Primary Health Care, Oulu University Hospital, OYS, Oulu, Finland; [35]Department of Life Sciences, College of Health and Life Sciences, Brunel University London, London, UK; [36]Research and Communication Unit for Musculoskeletal Health, Department of Research, Innovation and Education, Division of Clinical Neuroscience, Akershus University Hospital and University of Oslo, Oslo, Norway; [37]Department of General Practice, Institute of Health and Society, University of Oslo, Oslo, Norway; [38]Department of Neurology, Akershus University Hospital, Lørenskog, Norway; [39]Institute of Human Genetics, University Medical Center Hamburg-Eppendorf, Hamburg, Germany; [40]Psychiatric and Neurodevelopmental Genetics Unit, Department of Medicine, Massachusetts General Hospital, Boston, Massachusetts, USA; [41]Institute of Public Health, Charité – Universitätsmedizin, Berlin; [42]Laboratory of

Epidemiology and Population Sciences, Intramural Research Program, National Institute on Aging, Bethesda, Maryland, USA; [43]Department of Clinical Chemistry, Fimlab Laboratories, and Finnish Cardiovascular Research Center - Tampere, Faculty of Medicine and Health Technology, Tampere University, Tampere, Finland; [44]Max Planck Institute of Psychiatry, Munich, Germany; [45]23&Me Inc., Mountain View, California, USA; [46]School of Biomedical Sciences, Faculty of Health, Centre for Genomics and Personalised Health, Centre for Data Science, Queensland University of Technology, Brisbane, Queensland, Australia; [47]University of Helsinki, Helsinki, Finland; [48]Department of Research, Innovation and Education, Division of Clinical Neuroscience, Oslo University Hospital, Oslo, Norway; [49]Department of Medical Epidemiology and Biostatistics, Karolinska Institutet, Stockholm, Sweden; [50]Department of Mathematics and Statistics, University of Helsinki, Helsinki, Finland; [51]Department of Public Health, University of Helsinki, Helsinki, Finland; [52]Department of Complex Trait Genetics, Center for Neurogenomics and Cognitive Research, Neuroscience Campus Amsterdam, VU University, Amsterdam, The Netherlands; [53]Headache Unit, Neurology Department, Vall d'Hebron University Hospital, Barcelona, Spain; [54]Sutter Health, Sacramento, California, USA; [55]Centre for Population Health Research, University of Turku, Turku University Hospital, Turku, Finland; [56]Research Centre of Applied and Preventive Cardiovascular Medicine, University of Turku, Turku, Finland; [57]Department of Clinical Physiology and Nuclear Medicine, Turku University Hospital, Turku, Finland; [58]Department of Epidemiology, Erasmus University Medical Centre, Rotterdam, the Netherlands; [59]Department of Neurology, Oslo University Hospital, Oslo, Norway

Supplementary Table 1 Characteristics of included genetic instruments

SNP	chr	pos	EA	OA	Beta	se	p value	eaf	R^2	F statistic
rs6671730	1	2339139	G	A	0.0148	0.0020	1.92E-13	0.566	1.075E-04	54
rs35408430	1	17560195	C	T	0.0215	0.0021	1.36E-24	0.658	2.080E-04	105
rs7522116	1	41835685	C	T	0.0135	0.0020	2.97E-11	0.434	8.905E-05	44
rs11591147*	1	55505647	G	T	-0.0451	0.0075	1.64E-09	0.982	7.204E-05	36
rs2131925	1	63025942	G	T	0.0229	0.0021	3.61E-28	0.356	2.414E-04	121
rs7528419	1	109817192	A	G	-0.0197	0.0024	1.35E-16	0.775	1.358E-04	68
rs3768013	1	150815411	G	A	0.0132	0.0021	1.64E-10	0.631	8.172E-05	41
rs140371183	1	152098428	A	G	-0.0870	0.0057	1.86E-53	0.968	4.716E-04	237
rs12123821	1	152179152	C	T	-0.0786	0.0047	2.55E-63	0.952	5.587E-04	282
rs78886843	1	152412328	C	T	0.0253	0.0039	5.30E-11	0.928	8.537E-05	43
rs11264223	1	154565519	G	A	0.0123	0.0020	5.73E-10	0.510	7.619E-05	38
rs55668940*	1	155014426	G	A	0.0139	0.0025	2.17E-08	0.798	6.247E-05	31
rs7367758*	1	155087083	C	T	0.0175	0.0028	4.22E-10	0.849	7.825E-05	39
rs10908465	1	155389688	C	T	-0.0169	0.0022	6.12E-14	0.733	1.116E-04	56
rs867772	1	220972343	A	G	0.0146	0.0022	1.14E-11	0.315	9.211E-05	46
rs6672758	1	230303512	C	T	-0.0176	0.0025	2.40E-12	0.199	9.864E-05	49
rs7604788	2	21190024	C	T	-0.0337	0.0056	1.32E-09	0.967	7.329E-05	37
rs541041	2	21294975	G	A	0.0155	0.0026	2.27E-09	0.181	7.078E-05	36
rs1260326	2	27730940	T	C	-0.0206	0.0020	4.42E-24	0.393	2.028E-04	102
rs727857*	2	58981967	G	A	0.0140	0.0021	1.05E-11	0.389	9.337E-05	46
rs2710651	2	63166379	G	A	0.0115	0.0020	9.51E-09	0.472	6.542E-05	33
rs3888170*	2	101450679	A	G	0.0161	0.0026	8.03E-10	0.822	7.540E-05	38
rs7569755	2	118648261	G	A	-0.0143	0.0022	1.18E-10	0.709	8.372E-05	41
rs1047891*	2	211540507	C	A	0.0152	0.0021	1.18E-12	0.684	1.000E-04	51
rs2012736	2	234622379	C	A	0.0483	0.0037	1.16E-39	0.919	3.467E-04	174
rs6550617*	3	18777836	A	G	0.0124	0.0022	2.19E-08	0.282	6.245E-05	31
rs11721204	3	49982765	C	T	-0.0136	0.0020	1.17E-11	0.562	9.161E-05	46
rs6782190	3	85639672	G	A	0.0172	0.0021	1.45E-16	0.352	1.353E-04	68
rs7640441	3	125118082	C	A	-0.0132	0.0024	2.31E-08	0.763	6.320E-05	31
rs9861009	3	141654685	T	C	-0.0140	0.0023	4.86E-10	0.272	7.795E-05	39
rs73080940	4	3482296	T	C	0.0209	0.0033	1.63E-10	0.895	8.223E-05	41
rs4364259	4	15892159	G	A	-0.0159	0.0025	2.16E-10	0.798	8.167E-05	40
rs4616820*	4	57745481	C	T	0.0123	0.0020	1.13E-09	0.535	7.510E-05	37
rs35088884	4	69357050	A	C	-0.0117	0.0021	4.33E-08	0.343	6.133E-05	30
rs293435	4	69588052	C	T	0.0176	0.0022	1.59E-15	0.714	1.263E-04	64
rs7439366	4	69964338	T	C	0.0322	0.0020	1.41E-58	0.544	5.152E-04	260
rs71599974	4	71765339	A	G	-0.0260	0.0028	1.74E-20	0.852	1.709E-04	86
rs140090695	4	71879590	A	G	0.0408	0.0071	1.05E-08	0.980	6.657E-05	33
rs4130912	4	72322773	C	T	0.0240	0.0028	6.05E-18	0.846	1.496E-04	75
rs116778432	4	72360988	C	T	-0.0484	0.0077	3.26E-10	0.983	7.897E-05	40
rs113928144*	4	72428399	C	T	-0.0439	0.0078	2.03E-08	0.983	6.477E-05	31
rs144225950	4	72428624	T	C	0.0621	0.0089	3.53E-12	0.987	9.680E-05	48
rs4694421	4	72545947	T	G	-0.0314	0.0020	3.45E-55	0.553	4.888E-04	245
rs4694423	4	72554159	C	A	0.1007	0.0020	1.00E-200	0.584	4.933E-03	2482
rs705117	4	72608115	C	T	0.0334	0.0028	1.06E-32	0.148	2.812E-04	142
rs72645665	4	72703145	G	A	0.0886	0.0025	1.00E-200	0.809	2.423E-03	1219
rs139959724	4	72710250	A	G	-0.0874	0.0078	3.73E-29	0.983	2.517E-04	126
rs72648080	4	72732281	G	A	0.0758	0.0082	1.56E-20	0.985	1.721E-04	86
rs375491682	4	72741872	C	A	0.0771	0.0070	1.46E-28	0.021	2.437E-04	123
rs114296946	4	72757447	G	A	-0.0649	0.0094	3.95E-12	0.988	9.628E-05	48
rs113292111	4	72795966	C	T	-0.0990	0.0092	2.71E-27	0.988	2.353E-04	117
rs79484159	4	72819731	T	C	0.0454	0.0042	1.59E-27	0.940	2.339E-04	118
rs17775066	4	72834927	A	G	-0.0422	0.0068	4.54E-10	0.978	7.697E-05	39
rs114398069	4	72857132	G	A	-0.0666	0.0068	6.57E-23	0.978	1.929E-04	97
rs113209890	4	73236243	C	T	0.0540	0.0033	2.78E-61	0.897	5.394E-04	273
rs60321767	4	73543176	G	A	0.0458	0.0040	1.04E-29	0.935	2.546E-04	128
rs146332277	4	73756292	G	A	-0.0160	0.0022	9.90E-13	0.727	1.017E-04	51
rs55814693*	4	87401439	G	T	0.0130	0.0022	2.45E-09	0.700	7.093E-05	36
rs7691791	4	88286409	C	T	0.0160	0.0022	1.79E-13	0.702	1.076E-04	54
rs189407772	4	100146674	A	G	-0.0528	0.0067	3.90E-15	0.977	1.237E-04	62
rs1229984	4	100239319	T	C	0.0451	0.0064	1.53E-12	0.025	9.854E-05	50
rs66728779	4	100294444	C	T	0.0156	0.0025	4.68E-10	0.800	7.800E-05	39
rs10070734	5	87940026	T	C	-0.0132	0.0022	1.82E-09	0.290	7.197E-05	36
rs31612*	5	108996643	T	C	0.0145	0.0026	4.15E-08	0.826	6.079E-05	30
rs9325107	5	148016857	G	T	-0.0112	0.0020	3.23E-08	0.558	6.192E-05	31

rs72834856	6	22801858	T	G	0.0250	0.0039	8.67E-11	0.928	8.350E-05	42
rs9476310	6	57767576	C	T	-0.0118	0.0020	4.21E-09	0.489	6.908E-05	35
rs9490317*	6	121859499	T	C	-0.0111	0.0020	3.95E-08	0.554	6.035E-05	30
rs2248551	6	131924689	G	A	0.0234	0.0027	3.04E-18	0.835	1.506E-04	76
rs10085881	7	21577960	T	C	0.0146	0.0022	7.83E-11	0.718	8.585E-05	42
rs2862792	7	64032648	G	A	-0.0133	0.0020	3.98E-11	0.559	8.724E-05	44
rs11976765	7	100803242	C	T	0.0156	0.0028	3.38E-08	0.854	6.080E-05	30
rs6966728	7	104618318	C	T	0.0118	0.0020	8.01E-09	0.537	6.868E-05	33
rs1858889	7	107117447	A	C	-0.0112	0.0020	1.73E-08	0.498	6.312E-05	32
rs2346264	7	133536351	A	C	0.0139	0.0024	1.20E-08	0.217	6.556E-05	32
rs804281	8	11611865	A	G	-0.0133	0.0020	4.72E-11	0.416	8.597E-05	43
rs28692966	8	25892919	G	A	-0.0148	0.0023	1.13E-10	0.747	8.313E-05	42
rs4738684	8	59393273	A	G	-0.0124	0.0021	4.41E-09	0.334	6.857E-05	34
rs57163592	8	61332311	C	T	0.0175	0.0029	2.35E-09	0.867	7.091E-05	36
rs12056768	8	116988527	T	G	0.0234	0.0020	6.44E-31	0.417	2.663E-04	134
rs13284054*	9	107669073	T	C	-0.0176	0.0031	2.07E-08	0.882	6.412E-05	31
rs9409266	9	125745042	G	A	0.0196	0.0029	9.44E-12	0.139	9.221E-05	46
rs532436*	9	136149830	G	A	0.0185	0.0026	5.31E-13	0.816	1.031E-04	52
rs10887718*	10	82042624	C	T	0.0111	0.0020	2.61E-08	0.472	6.165E-05	31
rs77532868	10	88081438	C	T	-0.0266	0.0044	1.56E-09	0.946	7.218E-05	36
rs3925446	10	91495322	G	A	-0.0152	0.0025	1.09E-09	0.801	7.385E-05	37
rs4418728	10	94839724	G	T	-0.0110	0.0020	4.24E-08	0.548	5.951E-05	30
rs143645388	11	13350995	C	T	0.0523	0.0078	2.54E-11	0.983	8.893E-05	45
rs11022785	11	13411534	A	G	-0.0160	0.0028	1.56E-08	0.853	6.419E-05	32
rs138908802	11	13553360	C	T	-0.0214	0.0039	3.78E-08	0.929	6.039E-05	30
rs12361502	11	13942849	G	A	-0.0194	0.0025	2.74E-15	0.209	1.239E-04	62
rs12576218	11	14113228	G	A	0.0303	0.0042	3.42E-13	0.938	1.059E-04	53
rs543746207	11	14133656	A	G	0.3125	0.0088	1.00E-200	0.987	2.534E-03	1274
rs78708688	11	14410696	C	T	-0.0531	0.0078	1.31E-11	0.984	9.081E-05	46
rs150276002	11	14422316	G	A	-0.0433	0.0065	3.71E-11	0.976	8.697E-05	44
rs11023212	11	14431709	G	A	0.0715	0.0021	1.00E-200	0.656	2.309E-03	1140
rs11826356	11	14550516	C	T	-0.0745	0.0085	1.62E-18	0.986	1.544E-04	77
rs10160436	11	14570113	C	T	0.0628	0.0065	5.13E-22	0.976	1.852E-04	93
rs145382247	11	14613488	A	C	0.0575	0.0061	7.30E-21	0.973	1.747E-04	88
rs142881376	11	14623799	G	A	0.0563	0.0098	8.82E-09	0.989	6.591E-05	33
rs138600084	11	14632507	C	T	-0.0710	0.0081	1.57E-18	0.985	1.526E-04	77
rs118115876	11	14671396	G	A	-0.0730	0.0065	1.74E-29	0.976	2.531E-04	127
rs146128209	11	14683683	A	G	0.0575	0.0039	1.85E-49	0.929	4.357E-04	219
rs117247884	11	14729856	T	G	-0.0790	0.0086	2.81E-20	0.986	1.696E-04	85
rs117189917	11	14751650	G	A	-0.0626	0.0074	4.06E-17	0.982	1.405E-04	71
rs118010373	11	14757444	A	C	-0.0630	0.0053	2.14E-32	0.964	2.785E-04	140
rs118150101	11	14805350	G	T	-0.0516	0.0056	4.06E-20	0.967	1.678E-04	84
rs118010666	11	14860790	A	G	-0.0532	0.0054	1.19E-22	0.965	1.902E-04	96
rs117576073	11	14912573	G	T	0.1472	0.0089	5.91E-62	0.987	5.447E-04	276
rs59536295	11	14956391	C	T	-0.0415	0.0044	7.40E-21	0.947	1.737E-04	88
rs117079928	11	14966008	A	C	-0.0601	0.0042	8.34E-46	0.940	4.060E-04	202
rs61878675	11	14977461	C	T	-0.0640	0.0050	5.42E-37	0.959	3.198E-04	161
rs77486197	11	15059742	C	T	0.0540	0.0052	1.72E-25	0.962	2.158E-04	109
rs58149823*	11	15156949	A	G	-0.0506	0.0090	1.66E-08	0.988	6.313E-05	32
rs80240064	11	15290531	T	C	0.0356	0.0047	5.68E-14	0.953	1.129E-04	56
rs61880664	11	15343532	A	G	0.0216	0.0033	7.23E-11	0.898	8.485E-05	42
rs1872285	11	15665052	G	A	-0.0190	0.0025	1.04E-14	0.794	1.181E-04	60
rs1543832	11	15852684	A	C	0.0124	0.0021	4.30E-09	0.329	6.831E-05	34
rs76997982*	11	16674872	T	C	0.0210	0.0037	1.29E-08	0.921	6.459E-05	32
rs61891388	11	66079818	T	G	-0.0126	0.0020	4.06E-10	0.544	7.818E-05	39
rs71467497	11	70471414	T	C	0.0346	0.0060	8.40E-09	0.971	6.654E-05	33
rs78168201*	11	70971149	C	T	-0.0893	0.0086	5.18E-25	0.986	2.168E-04	107
rs75604577	11	71033252	C	A	0.0400	0.0070	1.31E-08	0.979	6.423E-05	32
rs57938057	11	71048671	T	C	0.0777	0.0076	1.00E-24	0.982	2.099E-04	105
rs1792329	11	71111182	C	T	-0.0261	0.0023	3.77E-29	0.745	2.585E-04	126
rs4944885	11	71114075	C	T	-0.0700	0.0021	1.00E-200	0.336	2.191E-03	1103
rs635421	11	71844612	C	T	-0.0285	0.0048	3.51E-09	0.046	7.084E-05	35
rs58411334	11	75467350	G	T	-0.0259	0.0034	4.60E-14	0.907	1.132E-04	57
rs1149605	11	76485216	T	C	-0.0220	0.0027	1.31E-16	0.830	1.370E-04	68
rs6589565	11	116640237	A	G	-0.0324	0.0040	4.80E-16	0.067	1.309E-04	66
rs12287066	11	116662331	G	T	0.0511	0.0041	1.06E-35	0.937	3.075E-04	156
rs2847500	11	120114421	G	A	0.0219	0.0030	4.42E-13	0.876	1.041E-04	52

rs11217815	11	120190262	G	A	0.0117	0.0021	1.30E-08	0.612	6.554E-05	32
rs12317268	12	21352541	A	G	0.0209	0.0028	6.19E-14	0.849	1.120E-04	56
rs11182428	12	38526387	T	C	0.0125	0.0020	3.23E-10	0.480	7.844E-05	40
rs1038165	12	68665940	C	T	-0.0121	0.0020	2.31E-09	0.417	7.066E-05	36
rs10859995	12	96375682	T	C	0.0403	0.0020	1.06E-88	0.417	7.917E-04	399
rs12307364	12	96382938	C	T	-0.0184	0.0023	4.27E-15	0.762	1.231E-04	62
rs12372115*	12	97982701	G	T	0.0218	0.0039	1.93E-08	0.929	6.244E-05	32
rs73413596	12	111582630	T	C	-0.0217	0.0038	1.41E-08	0.926	6.441E-05	32
rs9569209*	13	55707745	C	T	0.0127	0.0022	9.35E-09	0.714	6.530E-05	33
rs7149014*	14	29802911	T	C	0.0129	0.0021	5.42E-10	0.371	7.822E-05	39
rs2144530	14	39552484	C	T	0.0377	0.0026	2.99E-47	0.177	4.130E-04	208
rs4906378*	14	104283445	C	T	0.0128	0.0021	1.19E-09	0.661	7.372E-05	37
rs58038553	15	58574324	A	G	-0.0202	0.0030	1.48E-11	0.871	9.132E-05	46
rs261291	15	58680178	T	C	0.0274	0.0021	2.50E-39	0.645	3.430E-04	172
rs1800588	15	58723675	C	T	0.0329	0.0024	4.38E-42	0.785	3.661E-04	185
rs55829990	15	63790642	T	C	0.0186	0.0021	9.12E-19	0.656	1.562E-04	78
rs62007299	15	77711719	G	A	0.0133	0.0022	1.32E-09	0.287	7.291E-05	37
rs325384	15	100229761	C	T	0.0142	0.0022	1.66E-10	0.716	8.173E-05	41
rs7205121	16	11908776	T	C	-0.0134	0.0022	2.16E-09	0.726	7.123E-05	36
rs77924615	16	20392332	G	A	0.0166	0.0026	7.11E-11	0.807	8.633E-05	42
rs12928081	16	30885159	T	G	-0.0148	0.0023	5.61E-11	0.266	8.522E-05	43
rs11076175	16	57006378	A	G	-0.0230	0.0026	9.48E-19	0.822	1.557E-04	78
rs4327060*	16	72807438	C	T	0.0244	0.0044	2.92E-08	0.946	6.104E-05	31
rs4575545	16	79755446	G	A	0.0156	0.0022	7.47E-13	0.695	1.029E-04	51
rs11542462	16	82033810	G	A	0.0233	0.0029	1.27E-15	0.866	1.266E-04	64
rs10454087*	17	40735641	C	T	0.0135	0.0022	8.70E-10	0.715	7.459E-05	38
rs2952289	17	66464414	C	T	-0.0177	0.0025	1.18E-12	0.202	1.012E-04	51
rs8091117	18	28919794	C	A	0.0264	0.0040	5.98E-11	0.935	8.484E-05	43
rs77960347*	18	47109955	A	G	0.0494	0.0087	1.55E-08	0.987	6.332E-05	32
rs7244811	18	47156730	G	A	0.0164	0.0024	9.63E-12	0.227	9.447E-05	46
rs590215*	18	57904088	C	T	0.0129	0.0023	1.04E-08	0.734	6.519E-05	33
rs2037511	18	61366207	G	A	-0.0181	0.0027	1.35E-11	0.834	9.094E-05	46
rs142158911	19	11190534	G	A	-0.0255	0.0031	4.79E-16	0.885	1.323E-04	66
rs12462826	19	11955767	G	A	0.0124	0.0021	2.70E-09	0.631	7.129E-05	35
rs58542926	19	19379549	C	T	-0.0396	0.0038	6.87E-26	0.924	2.195E-04	111
rs187429064	19	19380513	A	G	-0.0648	0.0095	7.95E-12	0.989	9.364E-05	47
rs3814995	19	36342212	C	T	0.0126	0.0021	5.18E-09	0.688	6.766E-05	34
rs7412	19	45412079	C	T	-0.0300	0.0036	1.36E-16	0.918	1.360E-04	68
rs484195	19	45421877	A	G	0.0155	0.0021	1.37E-13	0.384	1.139E-04	55
rs212100	19	48376995	T	C	0.0662	0.0027	1.60E-133	0.164	1.200E-03	605
rs7248342	19	51515549	A	G	-0.0244	0.0024	7.30E-25	0.764	2.139E-04	106
rs8113404*	19	53065579	C	T	-0.0122	0.0022	2.07E-08	0.695	6.277E-05	31
rs60204587	19	54671421	G	A	0.0111	0.0020	4.66E-08	0.574	6.031E-05	30
rs2207132	20	39142516	G	A	0.0346	0.0056	5.56E-10	0.967	7.614E-05	38
rs6123359	20	52714706	A	G	-0.0342	0.0033	6.10E-25	0.898	2.145E-04	106
rs35194449	20	52742047	C	T	0.0375	0.0025	3.50E-50	0.805	4.408E-04	222
rs78851953	20	52783205	A	C	-0.0467	0.0064	3.13E-13	0.975	1.059E-04	53
rs2762943	20	52790786	T	G	-0.0457	0.0037	2.10E-34	0.077	2.969E-04	150
rs17274750*	21	16353809	A	C	0.0240	0.0034	8.90E-13	0.903	1.013E-04	51
rs6003465	22	23365501	T	C	0.0120	0.0021	1.77E-08	0.668	6.346E-05	32
rs3788428	22	31537533	A	G	-0.0254	0.0039	6.80E-11	0.929	8.467E-05	43
rs115621755	22	50853134	C	T	0.0124	0.0021	4.76E-09	0.673	6.803E-05	34

* SNPs excluded from Steiger-filtered analysis.

Abbreviations: EA, effect allele; eaf, effect allele frequency; OA other allele; SNP, single nucleotide polymorphism; R^2, proportion of variance explained.

Supplementary Table 2 SNP effects on migraine

SNP	chr	pos	EA	OA	Hautakangas et al. 2022			Choquet et al. 2021			FinnGen (Release 6)		
					Beta	se	p value	Beta	se	p value	Beta	se	p value
rs6671730	1	2339139	G	A	0.0047	0.0086	0.589	0.0096	0.0107	0.366	0.0247	0.0150	0.099
rs35408430	1	17560195	C	T	0.0033	0.0080	0.684	0.0068	0.0099	0.489	0.0052	0.0155	0.740
rs7522116	1	41835685	C	T	-0.0104	0.0078	0.179	0.0107	0.0089	0.231	-4.00E-05	0.0153	0.998
rs11591147	1	55505647	G	T	-0.0139	0.0334	0.679	NA	NA	NA	-0.0758	0.0394	0.054
rs2131925	1	63025942	G	T	-0.0143	0.0080	0.075	-0.0011	0.0096	0.909	0.0260	0.0170	0.127
rs7528419	1	109817192	A	G	0.0095	0.0092	0.298	0.0216	0.0110	0.049	0.0270	0.0183	0.140
rs3768013	1	150815411	G	A	0.0183	0.0080	0.022	0.0150	0.0092	0.101	-0.0209	0.0156	0.181
rs140371183	1	152098428	A	G	0.0483	0.0295	0.101	-0.0089	0.0265	0.738	-0.1159	0.0872	0.184
rs12123821	1	152179152	C	T	-0.0012	0.0219	0.956	-0.0033	0.0225	0.884	0.0004	0.0385	0.992
rs78886843	1	152412328	C	T	-0.0023	0.0169	0.894	-0.0236	0.0176	0.180	0.0127	0.0241	0.598
rs11264223	1	154565519	G	A	0.0020	0.0077	0.798	-0.0040	0.0088	0.651	-0.0063	0.0150	0.674
rs55668940	1	155014426	G	A	0.0120	0.0097	0.218	0.0110	0.0114	0.336	0.0064	0.0190	0.736
rs7367758	1	155087083	C	T	-0.0303	0.0103	0.003	-0.0217	0.0125	0.084	0.0194	0.0186	0.297
rs10908465	1	155389688	C	T	0.0047	0.0087	0.586	0.0120	0.0100	0.231	-0.0051	0.0166	0.757
rs867772	1	220972343	A	G	0.0039	0.0084	0.646	0.0096	0.0096	0.314	-0.0141	0.0165	0.393
rs6672758	1	230303512	C	T	0.0060	0.0095	0.525	0.0099	0.0109	0.361	-0.0186	0.0171	0.277
rs7604788	2	21190024	C	T	-0.0162	0.0203	0.426	-0.0085	0.0249	0.733	-0.0217	0.0313	0.488
rs541041	2	21294975	G	A	0.0070	0.0102	0.494	0.0069	0.0113	0.542	0.0202	0.0193	0.294
rs1260326	2	27730940	T	C	0.0123	0.0078	0.116	0.0238	0.0090	0.008	0.0254	0.0157	0.106
rs727857	2	58981967	G	A	0.0181	0.0078	0.020	0.0067	0.0090	0.456	0.0219	0.0150	0.145
rs2710651	2	63166379	G	A	-0.0062	0.0076	0.417	-0.0066	0.0088	0.456	0.0082	0.0151	0.587
rs3888170	2	101450679	A	G	-0.0051	0.0099	0.604	-0.0135	0.0111	0.224	-0.0355	0.0185	0.055
rs7569755	2	118648261	G	A	-0.0044	0.0085	0.601	-0.0177	0.0094	0.060	0.0042	0.0184	0.821
rs1047891	2	211540507	C	A	-0.0413	0.0084	8.22E-07	-0.0496	0.0094	1.49E-07	-0.0143	0.0160	0.371
rs2012736	2	234622379	C	A	-0.0073	0.0147	0.620	0.0156	0.0155	0.313	0.0062	0.0287	0.829
rs6550617	3	18777836	A	G	-0.0228	0.0084	0.007	-0.0179	0.0098	0.066	-0.0458	0.0158	0.004
rs11721204	3	49982765	C	T	-0.0151	0.0077	0.048	-0.0071	0.0091	0.438	-0.0140	0.0153	0.359
rs6782190	3	85639672	G	A	-0.0145	0.0080	0.070	-0.0286	0.0093	0.002	-0.0254	0.0169	0.133
rs7640441	3	125118082	C	A	0.0015	0.0088	0.863	-0.0166	0.0103	0.109	0.0264	0.0165	0.109
rs9861009	3	141654685	T	C	-0.0028	0.0086	0.745	-0.0036	0.0099	0.718	-0.0028	0.0171	0.872
rs73080940	4	3482296	T	C	-0.0152	0.0130	0.242	0.0169	0.0171	0.321	-0.0591	0.0228	0.010
rs4364259	4	15892159	G	A	0.0124	0.0103	0.231	-0.0060	0.0111	0.591	0.0185	0.0183	0.311
rs4616820	4	57745481	C	T	-0.0269	0.0076	4.40E-04	-0.0008	0.0089	0.928	-0.0266	0.0151	0.078
rs35088884	4	69357050	A	C	0.0163	0.0128	0.201	0.0070	0.0112	0.529	0.0016	0.0150	0.913
rs293435	4	69588052	C	T	0.0044	0.0102	0.664	0.0087	0.0097	0.367	0.0024	0.0152	0.876
rs7439366	4	69964338	T	C	-0.0023	0.0080	0.775	0.0111	0.0088	0.210	0.0061	0.0151	0.684

rs71599974	4	71765339	A	G	-0.0077	0.0114	0.497	0.0155	0.0125	0.214	-0.0154	0.0220	0.485
rs140090695	4	71879590	A	G	0.0296	0.0392	0.449	NA	NA	NA	0.0007	0.0370	0.984
rs4130912	4	72322773	C	T	0.0005	0.0106	0.960	-0.0013	0.0121	0.915	-0.0471	0.0231	0.042
rs116778432	4	72360988	C	T	0.0018	0.0348	0.960	NA	NA	NA	-0.0448	0.0542	0.408
rs113928144	4	72428399	C	T	-0.0164	0.0406	0.686	NA	NA	NA	0.1360	0.0804	0.091
rs144225950	4	72428624	T	C	0.0592	0.0588	0.314	0.0541	0.0398	0.174	-0.0687	0.0683	0.315
rs4694421	4	72545947	T	G	-0.0035	0.0076	0.645	0.0069	0.0090	0.441	0.0154	0.0151	0.308
rs4694423	4	72554159	C	A	-0.0071	0.0081	0.380	-0.0212	0.0091	0.019	-0.0422	0.0151	0.005
rs705117	4	72608115	C	T	0.0021	0.0106	0.843	-0.0128	0.0121	0.288	0.0464	0.0224	0.038
rs72645665	4	72703145	G	A	-0.0052	0.0096	0.587	-0.0042	0.0110	0.704	-0.0355	0.0185	0.055
rs139959724	4	72710250	A	G	0.0374	0.0362	0.301	0.0337	0.0364	0.355	-0.0151	0.0499	0.763
rs72648080	4	72732281	G	A	0.0209	0.0383	0.585	NA	NA	NA	0.0346	0.0496	0.485
rs375491682	4	72741872	C	A	NA	NA	NA	0.0231	0.0264	0.382	NA	NA	NA
rs114296946	4	72757447	G	A	0.0376	0.0610	0.538	0.0235	0.0406	0.563	0.0226	0.0714	0.751
rs113292111	4	72795966	C	T	0.0504	0.0335	0.132	NA	NA	NA	-0.0039	0.0521	0.941
rs79484159	4	72819731	T	C	-0.0257	0.0178	0.148	0.0109	0.0174	0.533	0.0277	0.0271	0.307
rs17775066	4	72834927	A	G	-0.0413	0.0296	0.163	-0.0066	0.0304	0.829	0.0789	0.0578	0.172
rs114398069	4	72857132	G	A	0.0511	0.0309	0.098	NA	NA	NA	-0.0308	0.0577	0.594
rs113209890	4	73236243	C	T	0.0060	0.0193	0.757	0.0003	0.0176	0.986	-0.0129	0.0270	0.633
rs60321767	4	73543176	G	A	0.0014	0.0153	0.928	0.0050	0.0174	0.773	-0.0021	0.0274	0.939
rs146332277	4	73756292	G	A	NA	NA	NA	-0.0068	0.0101	0.502	NA	NA	NA
rs55814693	4	87401439	G	T	-0.0079	0.0083	0.346	-0.0177	0.0097	0.068	0.0031	0.0166	0.854
rs7691791	4	88286409	C	T	-0.0084	0.0084	0.313	-0.0136	0.0095	0.153	0.0102	0.0172	0.553
rs189407772	4	100146674	A	G	0.0239	0.0290	0.410	0.0856	0.0322	0.008	0.0170	0.1024	0.868
rs1229984	4	100239319	T	C	0.0353	0.0265	0.183	0.0520	0.0217	0.016	0.0637	0.1056	0.547
rs66728779	4	100294444	C	T	-0.0113	0.0096	0.242	-0.0039	0.0107	0.716	-0.0272	0.0197	0.168
rs10070734	5	87940026	T	C	0.0075	0.0083	0.364	0.0092	0.0096	0.338	-0.0192	0.0169	0.255
rs31612	5	108996643	T	C	0.0140	0.0098	0.153	0.0105	0.0114	0.360	0.0043	0.0181	0.813
rs9325107	5	148016857	G	T	-0.0006	0.0078	0.934	-0.0159	0.0089	0.076	0.0058	0.0154	0.706
rs72834856	6	22801858	T	G	0.0137	0.0153	0.368	0.0171	0.0174	0.325	0.0187	0.0311	0.547
rs9476310	6	57767576	C	T	-0.0102	0.0079	0.193	0.0005	0.0092	0.957	-0.0107	0.0150	0.477
rs9490317	6	121859499	T	C	0.0217	0.0076	0.005	0.0237	0.0089	0.008	0.0151	0.0150	0.315
rs2248551	6	131924689	G	A	0.0201	0.0102	0.048	0.0364	0.0120	0.003	0.0162	0.0183	0.375
rs10085881	7	21577960	T	C	0.0016	0.0084	0.850	0.0042	0.0101	0.676	0.0212	0.0165	0.199
rs2862792	7	64032648	G	A	0.0036	0.0077	0.640	0.0060	0.0089	0.500	-0.0090	0.0152	0.552
rs11976765	7	100803242	C	T	-0.0119	0.0118	0.313	-0.0131	0.0122	0.282	0.0114	0.0211	0.590
rs6966728	7	104618318	C	T	-0.0077	0.0077	0.316	-0.0111	0.0090	0.214	-0.0015	0.0150	0.922
rs1858889	7	107117447	A	C	-0.0010	0.0076	0.894	0.0024	0.0090	0.789	-0.0130	0.0150	0.385
rs2346264	7	133536351	A	C	0.0002	0.0094	0.982	-0.0086	0.0111	0.441	0.0124	0.0206	0.547

rs804281	8	11611865	A	G	0.0025	0.0078	0.751	-0.0119	0.0090	0.187	-0.0030	0.0164	0.855
rs28692966	8	25892919	G	A	-0.0011	0.0086	0.896	0.0223	0.0101	0.028	-0.0034	0.0163	0.836
rs4738684	8	59393273	A	G	-0.0007	0.0081	0.934	-0.0055	0.0095	0.565	-0.0233	0.0155	0.131
rs57163592	8	61332311	C	T	0.0043	0.0110	0.694	-0.0029	0.0126	0.819	-0.0012	0.0198	0.951
rs12056768	8	116988527	T	G	-0.0070	0.0077	0.363	-0.0096	0.0090	0.287	-0.0176	0.0150	0.240
rs13284054	9	107669073	T	C	-0.0328	0.0121	0.007	-0.0050	0.0136	0.715	-0.0079	0.0243	0.744
rs9409266	9	125745042	G	A	0.0003	0.0108	0.977	0.0245	0.0123	0.047	-0.0137	0.0198	0.491
rs532436	9	136149830	G	A	-0.0272	0.0096	0.004	-0.0299	0.0112	0.008	-0.0279	0.0187	0.136
rs10887718	10	82042624	C	T	0.0154	0.0076	0.042	0.0052	0.0090	0.563	-0.0108	0.0152	0.475
rs77532868	10	88081438	C	T	-0.0042	0.0187	0.821	-0.0533	0.0203	0.009	-0.0260	0.0427	0.544
rs3925446	10	91495322	G	A	-0.0055	0.0095	0.561	0.0070	0.0109	0.521	0.0314	0.0166	0.058
rs4418728	10	94839724	G	T	-0.0016	0.0076	0.839	-0.0090	0.0089	0.315	0.0215	0.0150	0.151
rs143645388	11	13350995	C	T	0.0205	0.0359	0.568	NA	NA	NA	-0.0146	0.0437	0.738
rs11022785	11	13411534	A	G	-0.0166	0.0109	0.130	0.0096	0.0128	0.451	-0.0154	0.0226	0.497
rs138908802	11	13553360	C	T	0.0157	0.0153	0.305	-0.0013	0.0176	0.941	0.0017	0.0265	0.948
rs12361502	11	13942849	G	A	0.0105	0.0093	0.258	0.0311	0.0107	0.004	-0.0087	0.0201	0.665
rs12576218	11	14113228	G	A	0.0082	0.0155	0.595	0.0083	0.0173	0.631	-0.0110	0.0335	0.744
rs543746207	11	14133656	A	G	0.1010	0.0524	0.054	NA	NA	NA	NA	NA	NA
rs78708688	11	14410696	C	T	-0.0825	0.0348	0.018	NA	NA	NA	0.0115	0.1109	0.917
rs150276002	11	14422316	G	A	-0.0309	0.0250	0.217	0.0005	0.0302	0.987	-0.0728	0.0430	0.091
rs11023212	11	14431709	G	A	-0.0159	0.0080	0.047	-0.0283	0.0093	0.002	0.0226	0.0158	0.153
rs11826356	11	14550516	C	T	0.0248	0.0373	0.507	0.0371	0.0360	0.303	0.0271	0.0962	0.778
rs10160436	11	14570113	C	T	-0.0330	0.0292	0.258	-0.0168	0.0301	0.578	0.1002	0.0528	0.058
rs145382247	11	14613488	A	C	0.0190	0.0238	0.424	-0.0003	0.0278	0.991	-0.1177	0.0435	0.007
rs142881376	11	14623799	G	A	0.0256	0.0438	0.560	NA	NA	NA	-0.0697	0.0658	0.289
rs138600084	11	14632507	C	T	-0.0197	0.0357	0.582	NA	NA	NA	0.0521	0.0515	0.312
rs118115876	11	14671396	G	A	0.0384	0.0270	0.154	0.0661	0.0305	0.030	0.1691	0.0843	0.045
rs146128209	11	14683683	A	G	-0.0073	0.0154	0.637	0.0051	0.0162	0.753	-0.0004	0.0374	0.991
rs117247884	11	14729856	T	G	-0.0553	0.0339	0.103	-0.0282	0.0366	0.442	-0.0290	0.0688	0.673
rs117189917	11	14751650	G	A	0.0247	0.0319	0.438	-0.0039	0.0324	0.905	-0.0394	0.0532	0.459
rs118010373	11	14757444	A	C	-0.0174	0.0239	0.467	-0.0251	0.0250	0.316	0.0267	0.0455	0.557
rs118150101	11	14805350	G	T	0.0380	0.0235	0.106	0.0493	0.0259	0.057	0.0534	0.0407	0.190
rs118010666	11	14860790	A	G	0.0241	0.0223	0.280	-0.0439	0.0252	0.081	0.0092	0.0642	0.886
rs117576073	11	14912573	G	T	0.0398	0.0593	0.503	NA	NA	NA	-0.0030	0.0473	0.950
rs59536295	11	14956391	C	T	-0.0174	0.0194	0.368	-0.0299	0.0200	0.136	0.0193	0.0315	0.541
rs117079928	11	14966008	A	C	-0.0098	0.0157	0.534	-0.0051	0.0193	0.792	0.0171	0.0261	0.512
rs61878675	11	14977461	C	T	0.0354	0.0251	0.159	0.0649	0.0244	0.008	0.0672	0.0359	0.062
rs77486197	11	15059742	C	T	-0.0086	0.0205	0.674	-0.0544	0.0237	0.022	0.0285	0.0429	0.506
rs58149823	11	15156949	A	G	0.1313	0.0414	0.002	0.0232	0.0374	0.535	-0.0070	0.0580	0.905

rs80240064	11	15290531	T	C	-0.0197	0.0196	0.314	-0.0308	0.0195	0.114	0.0371	0.0448	0.407
rs61880664	11	15343532	A	G	-0.0143	0.0129	0.268	-0.0070	0.0147	0.636	-0.0366	0.0317	0.248
rs1872285	11	15665052	G	A	-0.0001	0.0094	0.989	-0.0184	0.0108	0.088	0.0047	0.0178	0.793
rs1543832	11	15852684	A	C	-0.0051	0.0080	0.521	0.0028	0.0095	0.769	0.0065	0.0156	0.676
rs76997982	11	16674872	T	C	0.0244	0.0169	0.149	0.0074	0.0169	0.661	0.0403	0.0376	0.284
rs61891388	11	66079818	T	G	0.0005	0.0076	0.952	0.0149	0.0093	0.108	0.0001	0.0150	0.994
rs71467497	11	70471414	T	C	0.0027	0.0210	0.897	-0.0281	0.0269	0.297	0.0371	0.0455	0.415
rs78168201	11	70971149	C	T	NA	NA	NA	NA	NA	NA	-0.2738	0.1152	0.018
rs75604577	11	71033252	C	A	NA	NA	NA	0.0063	0.0315	0.841	-0.0668	0.0414	0.106
rs57938057	11	71048671	T	C	-0.0975	0.0241	5.25E-05	-0.0498	0.0251	0.047	-0.0332	0.0346	0.336
rs1792329	11	71111182	C	T	0.0002	0.0089	0.984	0.0276	0.0102	0.007	0.0440	0.0185	0.017
rs4944885	11	71114075	C	T	0.0030	0.0080	0.705	0.0169	0.0093	0.068	0.0463	0.0151	0.002
rs635421	11	71844612	C	T	-0.0062	0.0254	0.807	-0.0015	0.0203	0.941	NA	NA	NA
rs58411334	11	75467350	G	T	-0.0171	0.0128	0.181	0.0051	0.0147	0.728	-0.0082	0.0245	0.737
rs1149605	11	76485216	T	C	-0.0097	0.0101	0.336	-0.0066	0.0119	0.579	-0.0108	0.0201	0.591
rs6589565	11	116640237	A	G	-0.0039	0.0149	0.795	0.0101	0.0168	0.550	0.0044	0.0278	0.876
rs12287066	11	116662331	G	T	0.0041	0.0159	0.798	0.0173	0.0179	0.332	-0.0410	0.0307	0.182
rs2847500	11	120114421	G	A	-0.0133	0.0119	0.266	-0.0027	0.0138	0.845	-0.0025	0.0213	0.907
rs11217815	11	120190262	G	A	0.0021	0.0079	0.788	-0.0082	0.0092	0.377	-0.0009	0.0151	0.954
rs12317268	12	21352541	A	G	-0.0165	0.0101	0.101	0.0076	0.0122	0.533	-0.0503	0.0167	0.003
rs11182428	12	38526387	T	C	-0.0006	0.0076	0.938	-0.0019	0.0089	0.830	0.0051	0.0150	0.734
rs1038165	12	68665940	C	T	0.0046	0.0077	0.548	0.0071	0.0090	0.426	-0.0135	0.0151	0.372
rs10859995	12	96375682	T	C	-0.0051	0.0077	0.510	-0.0032	0.0090	0.722	0.0025	0.0158	0.874
rs12307364	12	96382938	C	T	-0.0035	0.0088	0.694	0.0025	0.0103	0.807	0.0050	0.0168	0.765
rs12372115	12	97982701	G	T	0.0164	0.0156	0.293	0.0531	0.0174	0.002	-0.0087	0.0338	0.797
rs73413596	12	111582630	T	C	0.0017	0.0148	0.911	-0.0119	0.0162	0.460	-0.0176	0.0322	0.584
rs9569209	13	55707745	C	T	-0.0143	0.0084	0.088	0.0002	0.0107	0.985	-0.0128	0.0169	0.448
rs7149014	14	29802911	T	C	0.0145	0.0079	0.065	0.0324	0.0093	4.58E-04	0.0055	0.0152	0.720
rs2144530	14	39552484	C	T	0.0129	0.0101	0.198	0.0040	0.0116	0.730	-0.0161	0.0213	0.450
rs4906378	14	104283445	C	T	0.0120	0.0083	0.145	0.0176	0.0094	0.063	NA	NA	NA
rs58038553	15	58574324	A	G	0.0053	0.0115	0.645	-0.0015	0.0131	0.909	-0.0127	0.0253	0.616
rs261291	15	58680178	T	C	-0.0062	0.0080	0.435	-0.0122	0.0092	0.184	0.0198	0.0153	0.196
rs1800588	15	58723675	C	T	0.0077	0.0094	0.416	0.0147	0.0106	0.164	-0.0170	0.0173	0.324
rs55829990	15	63790642	T	C	-0.0018	0.0080	0.822	0.0007	0.0103	0.946	0.0190	0.0154	0.217
rs62007299	15	77711719	G	A	-0.0019	0.0083	0.816	-0.0159	0.0097	0.101	-0.0265	0.0160	0.098
rs325384	15	100229761	C	T	-0.0117	0.0085	0.171	0.0016	0.0099	0.872	-0.0079	0.0160	0.619
rs7205121	16	11908776	T	C	0.0028	0.0087	0.747	0.0066	0.0100	0.507	-0.0195	0.0166	0.241
rs77924615	16	20392332	G	A	-0.0014	0.0102	0.892	0.0099	0.0113	0.378	-0.0096	0.0182	0.597
rs12928081	16	30885159	T	G	-0.0117	0.0088	0.181	0.0074	0.0101	0.464	0.0028	0.0165	0.865

rs11076175	16	57006378	A	G	-0.0043	0.0100	0.665	-0.0112	0.0118	0.340	-0.0119	0.0200	0.552
rs4327060	16	72807438	C	T	-0.0086	0.0174	0.620	-0.0257	0.0170	0.132	-0.0166	0.0236	0.483
rs4575545	16	79755446	G	A	0.0068	0.0083	0.417	-0.0034	0.0096	0.723	0.0044	0.0163	0.785
rs11542462	16	82033810	G	A	-0.0003	0.0137	0.980	-0.0094	0.0136	0.490	-0.0002	0.0260	0.993
rs10454087	17	40735641	C	T	0.0244	0.0085	0.004	0.0313	0.0099	0.002	0.0234	0.0175	0.181
rs2952289	17	66464414	C	T	0.0030	0.0096	0.755	0.0058	0.0108	0.589	-0.0112	0.0195	0.567
rs8091117	18	28919794	C	A	-0.0042	0.0145	0.771	-0.0111	0.0167	0.505	0.0206	0.0279	0.461
rs77960347	18	47109955	A	G	0.0486	0.0359	0.175	NA	NA	NA	0.0038	0.0888	0.966
rs7244811	18	47156730	G	A	-0.0036	0.0089	0.686	0.0106	0.0104	0.312	0.0283	0.0166	0.088
rs590215	18	57904088	C	T	0.0159	0.0087	0.067	0.0312	0.0101	0.002	0.0162	0.0189	0.392
rs2037511	18	61366207	G	A	0.0077	0.0103	0.452	0.0157	0.0119	0.187	0.0356	0.0205	0.082
rs142158911	19	11190534	G	A	-0.0156	0.0124	0.209	-0.0279	0.0139	0.045	-0.0140	0.0245	0.567
rs12462826	19	11955767	G	A	-0.0021	0.0078	0.787	0.0012	0.0093	0.898	0.0065	0.0159	0.685
rs58542926	19	19379549	C	T	-0.0157	0.0144	0.276	-0.0027	0.0172	0.875	-0.0422	0.0306	0.168
rs187429064	19	19380513	A	G	-0.0228	0.0455	0.617	NA	NA	NA	-0.0029	0.0341	0.932
rs3814995	19	36342212	C	T	-0.0053	0.0093	0.572	-0.0058	0.0099	0.561	0.0169	0.0158	0.284
rs7412	19	45412079	C	T	0.0034	0.0145	0.818	-0.0159	0.0166	0.338	-0.0079	0.0337	0.814
rs484195	19	45421877	A	G	0.0019	0.0089	0.832	-0.0112	0.0112	0.316	-0.0112	0.0163	0.490
rs212100	19	48376995	T	C	0.0079	0.0105	0.452	0.0090	0.0122	0.459	0.0186	0.0208	0.373
rs7248342	19	51515549	A	G	0.0211	0.0093	0.024	0.0112	0.0109	0.307	0.0072	0.0173	0.678
rs8113404	19	53065579	C	T	-0.0077	0.0089	0.389	-0.0142	0.0097	0.143	-0.0063	0.0162	0.699
rs60204587	19	54671421	G	A	-0.0041	0.0081	0.616	0.0022	0.0110	0.841	0.0047	0.0156	0.764
rs2207132	20	39142516	G	A	-0.0243	0.0235	0.302	0.0045	0.0252	0.858	0.0351	0.0307	0.252
rs6123359	20	52714706	A	G	-0.0114	0.0130	0.378	0.0106	0.0142	0.457	-0.0004	0.0236	0.987
rs35194449	20	52742047	C	T	0.0189	0.0095	0.046	0.0121	0.0111	0.276	-0.0059	0.0178	0.741
rs78851953	20	52783205	A	C	0.0402	0.0238	0.090	0.0529	0.0292	0.070	-0.0386	0.0341	0.258
rs2762943	20	52790786	T	G	0.0026	0.0182	0.887	0.0240	0.0171	0.161	0.0118	0.0258	0.648
rs17274750	21	16353809	A	C	0.0242	0.0131	0.064	0.0261	0.0154	0.090	0.0147	0.0267	0.584
rs6003465	22	23365501	T	C	-0.0010	0.0081	0.906	-0.0109	0.0095	0.249	0.0006	0.0169	0.974
rs3788428	22	31537533	A	G	0.0121	0.0140	0.388	-0.0100	0.0162	0.534	0.0015	0.0213	0.943
rs115621755	22	50853134	C	T	-0.0031	0.0141	0.824	-0.0073	0.0099	0.461	0.0116	0.0152	0.445

Abbreviations: EA, effect allele; eaf, effect allele frequency; OA other allele; SNP, single nucleotide polymorphism.

Supplementary Table 3 SNP effects on migraine subtypes

SNP	chr	pos	EA	OA	Gormley et al. 2016						FinnGen (Release 6)					
					MA			MO			MA			MO		
					Beta	se	p value	Beta	se	p value	Beta	se	p value	Beta	se	p value
rs6671730	1	2339139	G	A	0.0495	0.0272	0.069	0.0141	0.0257	0.582	0.0463	0.0223	0.038	0.0281	0.0236	0.234
rs35408430	1	17560195	C	T	0.0396	0.0223	0.075	-0.0153	0.0201	0.447	-0.0090	0.0231	0.699	0.0154	0.0245	0.529
rs7522116	1	41835685	C	T	0.0191	0.0217	0.379	-0.0468	0.0197	0.018	0.0108	0.0229	0.637	-0.0154	0.0242	0.524
rs11591147	1	55505647	G	T	0.0484	0.0933	0.604	-0.1566	0.0915	0.087	-0.0814	0.0586	0.164	-0.1049	0.0618	0.090
rs2131925	1	63025942	G	T	0.0122	0.0224	0.588	-0.0127	0.0200	0.525	0.0323	0.0254	0.203	0.0143	0.0269	0.595
rs7528419	1	109817192	A	G	0.0583	0.0260	0.025	0.0501	0.0232	0.031	0.0382	0.0273	0.161	0.0350	0.0289	0.227
rs3768013	1	150815411	G	A	0.0012	0.0221	0.957	0.0563	0.0200	0.005	0.0039	0.0233	0.867	-0.0216	0.0247	0.381
rs140371183	1	152098428	A	G	0.2286	0.1321	0.083	-0.0444	0.1039	0.669	-0.0178	0.1315	0.892	-0.2392	0.1377	0.082
rs12123821	1	152179152	C	T	-0.0599	0.0636	0.346	-0.1979	0.0602	0.001	-0.0503	0.0573	0.380	0.0047	0.0607	0.938
rs78886843	1	152412328	C	T	-0.0300	0.0569	0.598	0.0295	0.0532	0.578	0.0310	0.0358	0.386	-0.0024	0.0379	0.950
rs11264223	1	154565519	G	A	0.0080	0.0209	0.702	0.0142	0.0190	0.457	0.0142	0.0224	0.526	-0.0147	0.0237	0.536
rs55668940	1	155014426	G	A	0.0185	0.0278	0.506	0.0438	0.0250	0.079	-0.0093	0.0284	0.743	-0.0046	0.0300	0.877
rs7367758	1	155087083	C	T	-0.0549	0.0285	0.054	-0.0269	0.0259	0.299	0.0125	0.0277	0.653	-0.0124	0.0294	0.674
rs10908465	1	155389688	C	T	-0.0354	0.0243	0.145	-0.0169	0.0220	0.441	-0.0303	0.0247	0.219	0.0186	0.0262	0.477
rs867772	1	220972343	A	G	-0.0069	0.0237	0.772	0.0090	0.0216	0.676	-0.0335	0.0246	0.174	-0.0011	0.0260	0.967
rs6672758	1	230303512	C	T	0.0300	0.0261	0.250	0.0201	0.0241	0.404	0.0058	0.0255	0.820	-0.0497	0.0271	0.066
rs7604788	2	21190024	C	T	-0.0327	0.0535	0.541	-0.0292	0.0504	0.563	-0.0013	0.0467	0.979	-0.0226	0.0494	0.647
rs541041	2	21294975	G	A	-0.0063	0.0277	0.821	-0.0114	0.0253	0.652	0.0197	0.0287	0.491	0.0824	0.0304	0.007
rs1260326	2	27730940	T	C	0.0036	0.0215	0.866	-0.0070	0.0194	0.718	0.0134	0.0234	0.567	0.0501	0.0248	0.043
rs727857	2	58981967	G	A	-0.0055	0.0214	0.799	0.0376	0.0194	0.052	0.0117	0.0224	0.601	0.0381	0.0237	0.108
rs2710651	2	63166379	G	A	-0.0100	0.0210	0.635	0.0364	0.0190	0.056	0.0076	0.0224	0.735	-0.0041	0.0238	0.862
rs3888170	2	101450679	A	G	-0.0578	0.0276	0.036	-0.0139	0.0253	0.583	-0.0004	0.0276	0.990	-0.0398	0.0292	0.173
rs7569755	2	118648261	G	A	0.0219	0.0235	0.350	0.0161	0.0211	0.445	-0.0177	0.0273	0.518	0.0461	0.0290	0.111
rs1047891	2	211540507	C	A	-0.0209	0.0239	0.381	-0.0296	0.0217	0.173	-0.0176	0.0239	0.462	-0.0133	0.0253	0.599
rs2012736	2	234622379	C	A	-0.0350	0.0451	0.437	-0.0423	0.0399	0.289	-0.0196	0.0428	0.647	0.0343	0.0453	0.449
rs6550617	3	18777836	A	G	-0.0374	0.0237	0.115	0.0089	0.0209	0.671	-0.0575	0.0235	0.014	-0.0105	0.0249	0.672
rs11721204	3	49982765	C	T	-0.0199	0.0216	0.358	-0.0027	0.0192	0.887	-0.0027	0.0228	0.908	-0.0401	0.0241	0.096
rs6782190	3	85639672	G	A	-0.0110	0.0229	0.632	0.0073	0.0200	0.715	-0.0466	0.0251	0.064	-0.0136	0.0266	0.608
rs7640441	3	125118082	C	A	0.0422	0.0245	0.085	-0.0145	0.0219	0.509	0.0333	0.0245	0.174	0.0467	0.0259	0.072
rs9861009	3	141654685	T	C	0.0025	0.0244	0.918	0.0052	0.0216	0.809	0.0106	0.0255	0.678	0.0137	0.0270	0.611
rs73080940	4	3482296	T	C	0.0128	0.0404	0.752	-0.0996	0.0370	0.007	-0.0262	0.0341	0.442	-0.1113	0.0360	0.002
rs4364259	4	15892159	G	A	0.0178	0.0311	0.566	0.0017	0.0276	0.951	0.0259	0.0272	0.340	0.0436	0.0288	0.130
rs4616820	4	57745481	C	T	0.0208	0.0216	0.335	-0.0808	0.0191	0.000	0.0010	0.0225	0.963	-0.0410	0.0237	0.084
rs35088884	4	69357050	A	C	NA	NA	NA	NA	NA	NA	0.0041	0.0224	0.854	0.0012	0.0237	0.959
rs293435	4	69588052	C	T	NA	NA	NA	NA	NA	NA	0.0039	0.0227	0.863	-0.0018	0.0240	0.940
rs7439366	4	69964338	T	C	-0.0106	0.0237	0.654	0.0039	0.0211	0.853	0.0448	0.0225	0.047	-0.0225	0.0239	0.345
rs71599974	4	71765339	A	G	-0.0335	0.0333	0.314	0.0263	0.0296	0.374	-0.0187	0.0329	0.570	-0.0115	0.0347	0.742
rs140090695	4	71879590	A	G	NA	NA	NA	NA	NA	NA	-0.0645	0.0546	0.238	0.0324	0.0583	0.579
rs4130912	4	72322773	C	T	-0.0389	0.0297	0.191	-0.0043	0.0263	0.870	-0.0824	0.0345	0.017	-0.0459	0.0365	0.209
rs116778432	4	72360988	C	T	0.0397	0.1163	0.733	-0.0232	0.1133	0.838	-0.2077	0.0804	0.010	-0.0203	0.0856	0.813
rs113928144	4	72428399	C	T	NA	NA	NA	NA	NA	NA	0.1453	0.1187	0.221	-0.0699	0.1259	0.579

rs144225950	4	72428624	T	C	NA	NA	NA	NA	NA	NA	0.0952	0.1020	0.350	-0.0209	0.1088	0.848
rs4694421	4	72545947	T	G	-0.0201	0.0215	0.350	-0.0204	0.0191	0.284	0.0307	0.0225	0.172	0.0300	0.0238	0.208
rs4694423	4	72554159	C	A	-0.0243	0.0242	0.316	-0.0171	0.0212	0.419	-0.0325	0.0226	0.150	-0.0475	0.0239	0.046
rs705117	4	72608115	C	T	0.0244	0.0303	0.421	0.0027	0.0263	0.917	0.0171	0.0335	0.609	0.0548	0.0355	0.122
rs72645665	4	72703145	G	A	-0.0270	0.0270	0.316	0.0054	0.0243	0.823	-0.0598	0.0276	0.030	-0.0451	0.0293	0.123
rs139959724	4	72710250	A	G	0.3067	0.1259	0.015	0.1820	0.1188	0.126	-0.0019	0.0742	0.979	-0.0124	0.0789	0.875
rs72648080	4	72732281	G	A	0.0310	0.1757	0.860	NA	NA	NA	0.0353	0.0740	0.633	-0.0333	0.0786	0.672
rs375491682	4	72741872	C	A	NA	NA	NA	NA	NA	NA	NA	NA	NA	NA	NA	NA
rs114296946	4	72757447	G	A	NA	NA	NA	NA	NA	NA	-0.0176	0.1064	0.868	0.0752	0.1127	0.505
rs113292111	4	72795966	C	T	0.1960	0.1114	0.079	0.1352	0.0976	0.166	-0.1094	0.0772	0.157	0.0894	0.0817	0.274
rs79484159	4	72819731	T	C	-0.0862	0.0538	0.109	-0.0062	0.0491	0.899	0.0766	0.0403	0.057	0.0014	0.0426	0.974
rs17775066	4	72834927	A	G	-0.0730	0.0907	0.420	-0.0740	0.0726	0.308	0.1192	0.0867	0.169	0.1022	0.0914	0.263
rs114398069	4	72857132	G	A	-0.0090	0.1080	0.934	0.0594	0.0948	0.531	0.0414	0.0866	0.633	-0.1023	0.0911	0.261
rs113209890	4	73236243	C	T	NA	NA	NA	NA	NA	NA	-0.0776	0.0402	0.054	0.0472	0.0427	0.269
rs60321767	4	73543176	G	A	-0.0299	0.0436	0.492	-0.0408	0.0392	0.298	-0.0263	0.0409	0.521	-0.0144	0.0433	0.740
rs146332277	4	73756292	G	A	NA	NA	NA	NA	NA	NA	NA	NA	NA	NA	NA	NA
rs55814693	4	87401439	G	T	-0.0242	0.0239	0.310	-0.0062	0.0212	0.769	-0.0084	0.0247	0.735	0.0368	0.0261	0.159
rs7691791	4	88286409	C	T	-0.0185	0.0237	0.435	0.0205	0.0210	0.329	-0.0138	0.0256	0.590	0.0366	0.0270	0.176
rs189407772	4	100146674	A	G	-0.0565	0.0817	0.489	0.0269	0.0687	0.696	0.0557	0.1522	0.714	-0.1015	0.1592	0.524
rs1229984	4	100239319	T	C	0.0223	0.0691	0.747	0.0282	0.0563	0.616	-0.0045	0.1591	0.977	-0.0137	0.1676	0.935
rs66728779	4	100294444	C	T	-0.0265	0.0275	0.335	-0.0246	0.0240	0.305	-0.0290	0.0293	0.324	-0.0631	0.0310	0.042
rs10070734	5	87940026	T	C	-0.0482	0.0238	0.043	0.0042	0.0207	0.840	-0.0108	0.0252	0.669	-0.0034	0.0267	0.898
rs31612	5	108996643	T	C	-0.0174	0.0272	0.522	0.0584	0.0248	0.018	0.0052	0.0269	0.848	-0.0046	0.0285	0.871
rs9325107	5	148016857	G	T	-0.0154	0.0219	0.482	-0.0050	0.0203	0.807	0.0197	0.0230	0.391	0.0156	0.0243	0.523
rs72834856	6	22801858	T	G	0.0263	0.0438	0.548	0.0170	0.0383	0.657	0.0409	0.0463	0.377	0.0223	0.0491	0.649
rs9476310	6	57767576	C	T	0.0029	0.0224	0.896	-0.0372	0.0204	0.069	0.0079	0.0224	0.726	-0.0308	0.0237	0.194
rs9490317	6	121859499	T	C	0.0255	0.0212	0.229	0.0479	0.0191	0.012	0.0134	0.0224	0.550	0.0107	0.0237	0.652
rs2248551	6	131924689	G	A	-0.0189	0.0279	0.498	-0.0192	0.0257	0.456	0.0078	0.0272	0.774	0.0184	0.0289	0.524
rs10085881	7	21577960	T	C	-0.0126	0.0242	0.604	-0.0032	0.0214	0.880	0.0297	0.0246	0.228	0.0319	0.0261	0.222
rs2862792	7	64032648	G	A	0.0066	0.0220	0.766	-0.0108	0.0195	0.580	0.0043	0.0226	0.850	-0.0178	0.0240	0.458
rs11976765	7	100803242	C	T	-0.0160	0.0308	0.605	-0.0249	0.0273	0.361	0.0294	0.0314	0.350	0.0117	0.0333	0.726
rs6966728	7	104618318	C	T	0.0093	0.0212	0.660	0.0048	0.0188	0.800	0.0228	0.0223	0.307	0.0273	0.0236	0.249
rs1858889	7	107117447	A	C	-0.0046	0.0214	0.831	-0.0004	0.0188	0.982	-0.0203	0.0224	0.365	-0.0080	0.0237	0.735
rs2346264	7	133536351	A	C	0.0036	0.0266	0.892	0.0142	0.0229	0.535	0.0445	0.0306	0.147	0.0509	0.0324	0.116
rs804281	8	11611865	A	G	-0.0244	0.0221	0.269	0.0317	0.0192	0.098	-0.0028	0.0244	0.910	0.0161	0.0259	0.533
rs28692966	8	25892919	G	A	0.0413	0.0245	0.092	-0.0081	0.0217	0.710	0.0010	0.0243	0.969	-0.0071	0.0258	0.782
rs4738684	8	59393273	A	G	-0.0010	0.0224	0.964	0.0257	0.0200	0.197	-0.0004	0.0230	0.988	-0.0264	0.0244	0.280
rs57163592	8	61332311	C	T	0.0228	0.0310	0.462	0.0241	0.0278	0.386	0.0425	0.0295	0.149	0.0146	0.0312	0.639
rs12056768	8	116988527	T	G	0.0063	0.0216	0.771	-0.0094	0.0192	0.624	0.0012	0.0224	0.957	-0.0310	0.0237	0.190
rs13284054	9	107669073	T	C	-0.0600	0.0346	0.083	-0.0354	0.0312	0.257	0.0147	0.0362	0.685	-0.0282	0.0383	0.462
rs9409266	9	125745042	G	A	-0.0083	0.0311	0.790	-0.0190	0.0280	0.497	-0.0244	0.0295	0.408	-0.0363	0.0313	0.246
rs532436	9	136149830	G	A	-0.0603	0.0265	0.023	0.0093	0.0241	0.698	-0.0693	0.0279	0.013	0.0116	0.0295	0.693
rs10887718	10	82042624	C	T	0.0079	0.0214	0.713	-0.0001	0.0189	0.996	0.0154	0.0226	0.494	-0.0138	0.0239	0.564
rs77532868	10	88081438	C	T	0.0890	0.0618	0.150	0.0588	0.0539	0.276	0.0638	0.0641	0.319	-0.0493	0.0674	0.465
rs3925446	10	91495322	G	A	-0.0269	0.0261	0.301	0.0381	0.0236	0.106	0.0304	0.0247	0.218	0.0075	0.0261	0.775

rs4418728	10	94839724	G	T	0.0149	0.0215	0.489	0.0349	0.0190	0.066	0.0645	0.0223	0.004	-0.0163	0.0236	0.489
rs143645388	11	13350995	C	T	-0.0798	0.1189	0.502	-0.0075	0.1275	0.953	-0.0400	0.0650	0.538	0.0816	0.0688	0.236
rs11022785	11	13411534	A	G	-0.0077	0.0306	0.801	-0.0172	0.0275	0.532	-0.0192	0.0338	0.569	-0.0383	0.0357	0.283
rs138908802	11	13553360	C	T	0.0081	0.0460	0.860	0.0169	0.0421	0.688	0.0055	0.0395	0.890	0.0032	0.0418	0.938
rs12361502	11	13942849	G	A	-0.0188	0.0259	0.467	0.0080	0.0230	0.727	-0.0202	0.0300	0.500	0.0124	0.0318	0.697
rs12576218	11	14113228	G	A	-0.0298	0.0415	0.472	0.0568	0.0377	0.132	-0.0229	0.0500	0.648	-0.0263	0.0530	0.620
rs543746207	11	14133656	A	G	NA	NA	NA	NA	NA	NA	NA	NA	NA	NA	NA	NA
rs78708688	11	14410696	C	T	-0.0373	0.1219	0.760	-0.1251	0.0995	0.209	-0.0805	0.1646	0.625	0.3259	0.1737	0.061
rs150276002	11	14422316	G	A	-0.0154	0.0731	0.833	-0.0795	0.0666	0.232	-0.1718	0.0642	0.007	-0.0791	0.0681	0.246
rs11023212	11	14431709	G	A	0.0024	0.0221	0.915	-0.0188	0.0199	0.347	0.0334	0.0235	0.157	0.0408	0.0249	0.101
rs11826356	11	14550516	C	T	-0.0203	0.1361	0.881	-0.0441	0.1208	0.715	-0.0227	0.1431	0.874	0.0488	0.1515	0.747
rs10160436	11	14570113	C	T	0.0582	0.1139	0.610	-0.1312	0.0927	0.157	0.0552	0.0786	0.483	0.0970	0.0832	0.244
rs145382247	11	14613488	A	C	-0.0389	0.0633	0.538	0.0231	0.0570	0.685	-0.0874	0.0648	0.177	-0.1283	0.0688	0.062
rs142881376	11	14623799	G	A	-0.1158	0.1272	0.362	0.1137	0.1287	0.377	-0.1016	0.0978	0.299	-0.0872	0.1042	0.403
rs138600084	11	14632507	C	T	-0.0583	0.1145	0.611	-0.0641	0.1058	0.545	0.1011	0.0761	0.184	0.0813	0.0808	0.314
rs118115876	11	14671396	G	A	0.0551	0.0752	0.464	-0.0007	0.0628	0.991	0.1446	0.1252	0.248	0.0845	0.1325	0.524
rs146128209	11	14683683	A	G	-0.0112	0.0443	0.801	-0.0550	0.0390	0.159	-0.0477	0.0558	0.393	0.0433	0.0592	0.465
rs117247884	11	14729856	T	G	0.0183	0.1025	0.858	0.0068	0.0941	0.943	-0.0942	0.1028	0.360	-0.0267	0.1088	0.806
rs117189917	11	14751650	G	A	0.1697	0.1048	0.106	0.1450	0.0994	0.145	-0.0580	0.0792	0.464	-0.0769	0.0840	0.360
rs118010373	11	14757444	A	C	-0.1457	0.0777	0.061	-0.0478	0.0749	0.524	0.0014	0.0682	0.983	0.1359	0.0720	0.059
rs118150101	11	14805350	G	T	0.0102	0.0716	0.887	0.0247	0.0642	0.701	0.0441	0.0605	0.467	0.0645	0.0640	0.314
rs118010666	11	14860790	A	G	-0.0390	0.0642	0.544	0.0774	0.0578	0.181	0.0070	0.0961	0.942	-0.0801	0.1006	0.426
rs117576073	11	14912573	G	T	NA	NA	NA	NA	NA	NA	0.0965	0.0704	0.170	-0.0636	0.0742	0.391
rs59536295	11	14956391	C	T	-0.0463	0.0613	0.450	-0.0197	0.0599	0.743	-0.0107	0.0469	0.819	-0.0699	0.0495	0.158
rs117079928	11	14966008	A	C	-0.0017	0.0422	0.968	-0.0577	0.0393	0.142	0.0386	0.0388	0.321	0.0270	0.0410	0.510
rs61878675	11	14977461	C	T	-0.1143	0.0851	0.179	0.1439	0.0895	0.108	0.0792	0.0533	0.138	0.0776	0.0565	0.170
rs77486197	11	15059742	C	T	-0.0562	0.0603	0.351	-0.0754	0.0535	0.159	0.0179	0.0643	0.781	-0.0034	0.0678	0.960
rs58149823	11	15156949	A	G	0.2005	0.1281	0.117	0.1748	0.1339	0.192	-0.0214	0.0863	0.804	-0.0732	0.0914	0.423
rs80240064	11	15290531	T	C	0.1274	0.0589	0.031	-0.0167	0.0505	0.741	-0.0423	0.0664	0.524	0.0636	0.0707	0.369
rs61880664	11	15343532	A	G	-0.0417	0.0365	0.252	0.0088	0.0325	0.787	-0.0383	0.0472	0.417	-0.0030	0.0500	0.952
rs1872285	11	15665052	G	A	-0.0231	0.0258	0.371	0.0124	0.0238	0.603	0.0353	0.0266	0.184	0.0349	0.0282	0.215
rs1543832	11	15852684	A	C	-0.0146	0.0222	0.509	0.0003	0.0200	0.987	0.0027	0.0232	0.909	0.0018	0.0246	0.942
rs76997982	11	16674872	T	C	0.0119	0.0556	0.831	0.0591	0.0515	0.251	0.0079	0.0560	0.888	0.0134	0.0592	0.821
rs61891388	11	66079818	T	G	-0.0424	0.0211	0.045	-0.0268	0.0192	0.162	-0.0129	0.0223	0.562	0.0219	0.0236	0.353
rs71467497	11	70471414	T	C	-0.0203	0.0571	0.722	0.0762	0.0528	0.148	0.0664	0.0678	0.327	0.0147	0.0716	0.837
rs78168201	11	70971149	C	T	NA	NA	NA	NA	NA	NA	-0.2708	0.1739	0.119	-0.3124	0.1854	0.092
rs75604577	11	71033252	C	A	-0.1067	0.0697	0.126	0.0484	0.0681	0.477	-0.0080	0.0616	0.897	-0.0253	0.0654	0.699
rs57938057	11	71048671	T	C	-0.1028	0.0626	0.101	-0.1382	0.0601	0.021	-0.0063	0.0514	0.903	-0.1027	0.0545	0.060
rs1792329	11	71111182	C	T	-0.0074	0.0251	0.768	-0.0485	0.0224	0.030	0.0433	0.0276	0.117	0.0345	0.0292	0.238
rs4944885	11	71114075	C	T	-0.0169	0.0219	0.439	0.0219	0.0197	0.265	0.0214	0.0224	0.340	0.0462	0.0238	0.052
rs635421	11	71844612	C	T	-0.0249	0.0716	0.728	0.0017	0.0690	0.980	NA	NA	NA	NA	NA	NA
rs58411334	11	75467350	G	T	-0.0006	0.0350	0.987	0.0007	0.0321	0.982	-0.0349	0.0365	0.338	-0.0122	0.0386	0.752
rs1149605	11	76485216	T	C	-0.0633	0.0283	0.025	-0.0044	0.0258	0.864	0.0196	0.0300	0.514	-0.0289	0.0317	0.361
rs6589565	11	116640237	A	G	-0.0034	0.0414	0.935	-0.0153	0.0371	0.680	0.0151	0.0415	0.716	0.0097	0.0439	0.826
rs12287066	11	116662331	G	T	-0.0199	0.0437	0.649	0.0077	0.0399	0.847	0.0159	0.0458	0.728	-0.0366	0.0486	0.451

rs2847500	11	120114421	G	A	0.0012	0.0350	0.972	-0.0335	0.0313	0.284	-0.0247	0.0317	0.437	0.0259	0.0336	0.440
rs11217815	11	120190262	G	A	-0.0034	0.0219	0.875	-0.0054	0.0200	0.787	-0.0106	0.0226	0.638	-0.0167	0.0239	0.484
rs12317268	12	21352541	A	G	0.0065	0.0274	0.813	-0.0203	0.0253	0.423	-0.0314	0.0248	0.206	-0.0390	0.0263	0.138
rs11182428	12	38526387	T	C	0.0193	0.0214	0.366	-0.0141	0.0192	0.463	0.0167	0.0224	0.456	0.0061	0.0237	0.796
rs1038165	12	68665940	C	T	0.0458	0.0213	0.032	-0.0180	0.0192	0.348	-0.0065	0.0225	0.772	-0.0374	0.0239	0.117
rs10859995	12	96375682	T	C	-0.0043	0.0212	0.839	0.0056	0.0191	0.771	-0.0009	0.0236	0.971	-0.0181	0.0250	0.468
rs12307364	12	96382938	C	T	0.0001	0.0239	0.996	-0.0070	0.0218	0.748	-0.0038	0.0250	0.879	-0.0043	0.0265	0.871
rs12372115	12	97982701	G	T	-0.0192	0.0449	0.669	0.0016	0.0401	0.967	0.0071	0.0504	0.888	-0.0275	0.0534	0.607
rs73413596	12	111582630	T	C	-0.0619	0.0422	0.142	-0.0109	0.0378	0.773	0.0266	0.0481	0.580	-0.0232	0.0507	0.648
rs9569209	13	55707745	C	T	-0.0115	0.0234	0.622	-0.0615	0.0210	0.003	0.0011	0.0251	0.965	-0.0242	0.0266	0.363
rs7149014	14	29802911	T	C	0.0057	0.0217	0.794	-0.0128	0.0198	0.518	-0.0018	0.0227	0.935	0.0407	0.0240	0.090
rs2144530	14	39552484	C	T	0.0021	0.0284	0.942	-0.0297	0.0256	0.247	-0.0103	0.0317	0.746	0.0362	0.0336	0.282
rs4906378	14	104283445	C	T	-0.0169	0.0246	0.492	0.0095	0.0219	0.664	NA	NA	NA	NA	NA	NA
rs58038553	15	58574324	A	G	-0.0383	0.0321	0.233	0.0168	0.0289	0.562	-0.0207	0.0377	0.583	0.0088	0.0400	0.827
rs261291	15	58680178	T	C	-0.0299	0.0222	0.178	-0.0195	0.0201	0.333	-0.0079	0.0229	0.730	0.0345	0.0242	0.153
rs1800588	15	58723675	C	T	0.0100	0.0257	0.697	0.0217	0.0236	0.359	-0.0372	0.0257	0.149	-0.0240	0.0272	0.377
rs55829990	15	63790642	T	C	-0.0187	0.0219	0.393	0.0003	0.0200	0.986	0.0334	0.0229	0.144	-0.0148	0.0242	0.543
rs62007299	15	77711719	G	A	0.0181	0.0230	0.433	-0.0238	0.0208	0.253	-0.0268	0.0238	0.260	-0.0192	0.0252	0.446
rs325384	15	100229761	C	T	-0.0506	0.0251	0.044	0.0023	0.0224	0.917	-0.0161	0.0238	0.497	-0.0395	0.0252	0.116
rs7205121	16	11908776	T	C	-0.0369	0.0244	0.131	-0.0151	0.0221	0.495	-0.0085	0.0247	0.733	-0.0595	0.0261	0.023
rs77924615	16	20392332	G	A	-0.0071	0.0285	0.803	0.0205	0.0267	0.442	-0.0006	0.0272	0.983	0.0301	0.0288	0.296
rs12928081	16	30885159	T	G	-0.0700	0.0242	0.004	-0.0166	0.0218	0.446	-0.0362	0.0246	0.141	0.0404	0.0260	0.120
rs11076175	16	57006378	A	G	-0.0252	0.0282	0.371	0.0349	0.0254	0.168	0.0223	0.0299	0.455	-0.0236	0.0316	0.455
rs4327060	16	72807438	C	T	-0.0591	0.0499	0.236	0.0148	0.0509	0.772	-0.0132	0.0352	0.706	0.0019	0.0372	0.960
rs4575545	16	79755446	G	A	0.0137	0.0231	0.553	0.0122	0.0211	0.563	0.0088	0.0243	0.717	0.0071	0.0257	0.781
rs11542462	16	82033810	G	A	-0.0577	0.0417	0.166	0.0077	0.0393	0.845	-0.0243	0.0388	0.530	0.0285	0.0410	0.486
rs10454087	17	40735641	C	T	0.0195	0.0236	0.410	0.0100	0.0211	0.635	0.0260	0.0261	0.319	0.0306	0.0277	0.269
rs2952289	17	66464414	C	T	0.0067	0.0266	0.802	0.0391	0.0239	0.102	0.0082	0.0291	0.779	-0.0190	0.0308	0.536
rs8091117	18	28919794	C	A	-0.0985	0.0387	0.011	-0.0169	0.0360	0.638	-0.0181	0.0414	0.663	0.0235	0.0439	0.593
rs77960347	18	47109955	A	G	0.0360	0.1145	0.753	0.0440	0.0892	0.622	0.0961	0.1330	0.470	-0.0186	0.1409	0.895
rs7244811	18	47156730	G	A	-0.0121	0.0248	0.627	-0.0205	0.0228	0.370	0.0285	0.0247	0.250	0.0328	0.0262	0.210
rs590215	18	57904088	C	T	-0.0056	0.0238	0.813	0.0193	0.0211	0.360	0.0102	0.0282	0.718	0.0175	0.0299	0.557
rs2037511	18	61366207	G	A	-0.0536	0.0280	0.056	-0.0047	0.0253	0.853	0.0481	0.0304	0.114	0.0281	0.0322	0.383
rs142158911	19	11190534	G	A	-0.0746	0.0345	0.030	0.0317	0.0324	0.329	0.0200	0.0364	0.584	-0.0216	0.0384	0.574
rs12462826	19	11955767	G	A	-0.0043	0.0216	0.842	-0.0156	0.0193	0.420	-0.0237	0.0237	0.318	-0.0129	0.0251	0.608
rs58542926	19	19379549	C	T	-0.0219	0.0420	0.603	0.0117	0.0378	0.757	-0.0689	0.0457	0.132	-0.0086	0.0483	0.860
rs187429064	19	19380513	A	G	NA	NA	NA	NA	NA	NA	-0.0290	0.0507	0.567	0.0174	0.0538	0.747
rs3814995	19	36342212	C	T	0.0187	0.0267	0.484	-0.0151	0.0245	0.538	-0.0006	0.0235	0.980	0.0473	0.0249	0.058
rs7412	19	45412079	C	T	0.1263	0.0432	0.003	-0.0344	0.0370	0.353	0.0472	0.0505	0.350	-0.0558	0.0533	0.295
rs484195	19	45421877	A	G	0.0208	0.0231	0.368	0.0381	0.0208	0.068	0.0013	0.0243	0.957	0.0183	0.0257	0.476
rs212100	19	48376995	T	C	-0.0038	0.0293	0.896	0.0258	0.0262	0.325	0.0208	0.0310	0.502	0.0123	0.0328	0.708
rs7248342	19	51515549	A	G	-0.0084	0.0261	0.749	0.0094	0.0242	0.696	0.0153	0.0257	0.552	0.0503	0.0273	0.065
rs8113404	19	53065579	C	T	-0.0173	0.0264	0.514	-0.0341	0.0248	0.170	-0.0300	0.0241	0.214	0.0229	0.0256	0.370
rs60204587	19	54671421	G	A	-0.0105	0.0242	0.664	0.0068	0.0216	0.752	0.0288	0.0232	0.214	-0.0251	0.0246	0.307
rs2207132	20	39142516	G	A	0.0108	0.0831	0.897	-0.1558	0.0805	0.053	0.0396	0.0456	0.386	0.0298	0.0483	0.537

rs6123359	20	52714706	A	G	-0.0113	0.0354	0.749	0.0334	0.0329	0.310	-0.0298	0.0351	0.397	-0.0545	0.0373	0.144
rs35194449	20	52742047	C	T	0.0311	0.0259	0.230	0.0100	0.0233	0.667	0.0009	0.0266	0.974	-0.0001	0.0281	0.998
rs78851953	20	52783205	A	C	0.0183	0.0632	0.772	-0.0432	0.0585	0.460	-0.0137	0.0508	0.787	-0.0947	0.0537	0.078
rs2762943	20	52790786	T	G	-0.0110	0.0672	0.870	-0.0524	0.0688	0.447	0.0198	0.0385	0.606	-0.0139	0.0408	0.733
rs17274750	21	16353809	A	C	0.0167	0.0357	0.639	0.0337	0.0322	0.295	0.0708	0.0399	0.076	0.0517	0.0424	0.222
rs6003465	22	23365501	T	C	-0.0215	0.0227	0.344	0.0099	0.0203	0.625	0.0149	0.0252	0.555	-0.0239	0.0267	0.371
rs3788428	22	31537533	A	G	0.0603	0.0376	0.109	-0.0227	0.0343	0.509	-0.0053	0.0316	0.867	0.0169	0.0335	0.615
rs115621755	22	50853134	C	T	0.0249	0.0241	0.300	-0.0062	0.0213	0.770	0.0202	0.0227	0.373	0.0317	0.0240	0.186

Abbreviations: EA, effect allele; eaf, effect allele frequency; MA, migraine with aura; MO, migraine without aura; OA other allele; SNP, single nucleotide polymorphism.

Supplementary Table 4 Potential pleiotropic variants and their effects on confounders

SNP	Effect allele	Other allele	Beta	se	Coufounders	p value
rs1047891	A	C	-0.1405	0.0188	Diastolic blood pressure	8.19E-14
rs11022785	G	A	-0.0213	0.0035	Serum calcium	1.40E-09
rs11023212	A	G	0.1110	0.0182	Diastolic blood pressure	1.04E-09
rs1229984	C	T	0.1881	0.0062	Drinking	1.60E-203
rs1260326	C	T	0.0238	0.0020	Drinking	3.33E-33
rs1260326	C	T	-0.0505	0.0026	Serum calcium	1.94E-86
rs1260326	C	T	0.0130	0.0016	Coffee consumption	3.60E-15
rs12928081	T	G	0.1552	0.0198	Diastolic blood pressure	4.60E-15
rs1858889	C	A	-0.0181	0.0025	Serum calcium	3.13E-13
rs2762943	G	T	0.0554	0.0047	Serum calcium	9.96E-32
rs28692966	A	G	-0.1917	0.0199	Diastolic blood pressure	4.74E-22
rs35194449	T	C	-0.0580	0.0031	Serum calcium	6.74E-77
rs532436	A	G	-0.2821	0.0224	Diastolic blood pressure	1.68E-36
rs58542926	T	C	0.0336	0.0047	Serum calcium	1.03E-12
rs58542926	T	C	0.2438	0.0334	Diastolic blood pressure	2.66E-13
rs590215	T	C	0.0147	0.0018	Coffee consumption	8.60E-16
rs6123359	G	A	0.0443	0.0041	Serum calcium	1.20E-27
rs66728779	T	C	-0.0178	0.0024	Drinking	2.18E-13
rs6782190	A	G	-0.0145	0.0020	Drinking	8.89E-13
rs6782190	A	G	0.1074	0.0181	Diastolic blood pressure	3.18E-09
rs6782190	A	G	-0.0300	0.0037	Smoking	9.87E-16
rs727857	G	A	0.0086	0.0014	Insomnia	1.50E-09
rs73080940	C	T	0.0348	0.0040	Serum calcium	2.72E-18
rs73413596	C	T	-0.2147	0.0333	Diastolic blood pressure	1.15E-10
rs77924615	A	G	-0.3163	0.0224	Diastolic blood pressure	3.72E-45
rs78851953	C	A	0.0567	0.0079	Serum calcium	8.61E-13
rs804281	G	A	0.1080	0.0177	Diastolic blood pressure	1.02E-09

Supplementary Table 5 SNPs for reverse Mendelian Randomization analysis

SNP	chr	pos	EA	OA	Migraine, Hautakangas et al. 2022				Vitamin D		
					Beta	se	eaf	p value	beta	se	p value
rs2124663	1	2833427	T	C	-0.0375	0.0063	0.222	2.44E-09	2.159E-04	2.422E-03	0.929
rs10218452	1	3075597	G	A	0.1100	0.0062	0.229	7.26E-71	1.978E-03	2.388E-03	0.408
rs2483262	1	3229134	T	G	0.0483	0.0060	0.232	1.36E-15	1.553E-03	2.388E-03	0.515
rs10128028	1	7055843	C	T	-0.0298	0.0052	0.481	7.66E-09	-2.717E-03	2.002E-03	0.175
rs12057629	1	15538493	C	T	0.0400	0.0054	0.356	9.38E-14	9.677E-04	2.092E-03	0.644
rs28739509	1	38366907	C	T	0.0386	0.0061	0.273	2.64E-10	-8.037E-04	2.229E-03	0.718
rs1472662*	1	39590409	T	G	0.0352	0.0062	0.216	1.75E-08	-6.641E-03	2.439E-03	0.006
rs11578492	1	60529980	C	A	0.0314	0.0054	0.420	6.25E-09	1.100E-03	2.048E-03	0.591
rs7511672	1	66178918	A	G	-0.0313	0.0052	0.462	1.43E-09	-2.064E-03	2.001E-03	0.302
rs4463622	1	73757456	A	G	-0.0383	0.0054	0.497	1.29E-12	-2.754E-03	2.011E-03	0.171
rs11165300*	1	92177663	G	T	0.0329	0.0060	0.239	4.72E-08	-5.040E-03	2.338E-03	0.031
rs2078371	1	115677183	C	T	0.1062	0.0078	0.117	5.87E-42	-8.082E-03	3.125E-03	0.010
rs11102915	1	115820598	C	T	-0.0436	0.0054	0.364	4.10E-16	1.311E-03	2.075E-03	0.527
rs68002561	1	149880863	G	A	0.0591	0.0094	0.089	3.61E-10	8.173E-03	3.566E-03	0.022
rs6693567	1	150510660	C	T	0.0435	0.0059	0.269	1.25E-13	-3.885E-03	2.249E-03	0.084
rs2274319	1	156450873	T	C	0.0727	0.0054	0.347	2.74E-41	1.541E-03	2.094E-03	0.462
rs2272785	1	175161599	T	G	-0.0324	0.0058	0.270	2.36E-08	-3.406E-04	2.247E-03	0.880
rs6668908	1	186913055	T	G	-0.0307	0.0055	0.331	2.22E-08	2.968E-03	2.109E-03	0.159
rs56140113	1	206843108	T	C	-0.0368	0.0064	0.220	7.76E-09	1.757E-03	2.392E-03	0.463
rs72764846	1	245847455	A	G	-0.0375	0.0064	0.216	5.41E-09	3.947E-04	2.405E-03	0.870
rs12712881	2	43649780	A	C	0.0328	0.0052	0.435	3.50E-10	-1.406E-03	2.018E-03	0.486
rs62153692	2	96569017	T	C	-0.0347	0.0058	0.329	2.65E-09	9.195E-04	2.159E-03	0.670
rs7564469*	2	145258445	C	T	0.0412	0.0071	0.161	5.06E-09	-7.674E-03	2.752E-03	0.005
rs895219	2	146037564	C	T	0.0370	0.0056	0.300	3.74E-11	-4.184E-03	2.171E-03	0.054
rs843215	2	156416638	G	A	0.0287	0.0051	0.468	2.61E-08	-1.728E-03	2.007E-03	0.389
rs72923449*	2	176978383	C	A	0.0777	0.0142	0.036	4.66E-08	1.251E-02	5.252E-03	0.017
rs138556413	2	203832867	T	C	-0.1293	0.0159	0.036	4.15E-16	9.242E-03	5.194E-03	0.075
rs10166942	2	234825093	C	T	-0.0992	0.0066	0.195	9.35E-51	2.072E-03	2.514E-03	0.410
rs7371912	3	30472786	A	G	0.0440	0.0057	0.303	1.06E-14	-5.294E-04	2.152E-03	0.806
rs950570	3	80302512	T	C	0.0567	0.0100	0.071	1.30E-08	-4.373E-03	3.702E-03	0.238
rs6795209	3	88210464	A	G	0.0413	0.0072	0.191	1.23E-08	1.928E-03	2.782E-03	0.488
rs1499963	3	124607055	T	C	-0.0321	0.0056	0.321	7.48E-09	-6.054E-04	2.138E-03	0.777
rs13078967	3	154289946	C	A	-0.1460	0.0178	0.027	2.16E-16	-1.446E-04	6.651E-03	0.983
rs58295024	4	35551447	A	C	-0.0430	0.0071	0.171	1.14E-09	-1.098E-03	2.585E-03	0.671
rs7684253*	4	57727311	C	T	-0.0392	0.0052	0.450	4.21E-14	1.003E-02	2.022E-03	7.10E-07
rs145639541	5	74994913	C	T	0.0414	0.0060	0.295	4.30E-12	4.889E-03	2.162E-03	0.024
rs12653216*	5	81129663	T	C	0.0370	0.0064	0.212	8.08E-09	-4.523E-03	2.479E-03	0.068
rs11957829	5	121515195	G	A	0.0411	0.0068	0.172	1.58E-09	-1.162E-03	2.628E-03	0.658
rs246326	5	122306398	T	C	0.0476	0.0077	0.126	6.80E-10	2.359E-03	3.057E-03	0.440
rs10038882	5	145752008	C	T	-0.0425	0.0060	0.252	1.33E-12	-3.454E-03	2.280E-03	0.130
rs4705403	5	149380493	A	G	0.0471	0.0083	0.104	1.18E-08	1.247E-03	3.190E-03	0.696
rs6556059	5	172645766	T	C	0.0336	0.0055	0.363	8.16E-10	1.624E-03	2.090E-03	0.437
rs35288939	5	176533852	A	G	-0.0410	0.0075	0.155	4.78E-08	3.042E-03	2.898E-03	0.294
rs9349379	6	12903957	G	A	-0.0772	0.0053	0.410	1.41E-47	2.837E-03	2.027E-03	0.162
rs9295536	6	22131929	A	C	-0.0355	0.0052	0.438	7.75E-12	-2.669E-04	2.008E-03	0.894
rs74434374	6	31850308	A	C	-0.0737	0.0126	0.051	4.52E-09	-8.768E-03	4.861E-03	0.071
rs10456100	6	39183470	T	C	0.0507	0.0057	0.281	9.16E-19	8.862E-04	2.210E-03	0.688
rs34273564	6	72321017	T	C	0.0336	0.0052	0.481	1.00E-10	-2.927E-03	2.022E-03	0.148
rs12524072	6	96568381	A	G	0.0306	0.0054	0.349	1.63E-08	6.826E-05	2.092E-03	0.974
rs11153082	6	97059666	G	A	0.0840	0.0054	0.331	7.26E-54	-2.321E-03	2.135E-03	0.277
rs6568677	6	111713302	A	G	0.0352	0.0063	0.209	2.09E-08	-3.409E-03	2.480E-03	0.169
rs28455731	6	121846038	T	G	0.0690	0.0070	0.157	8.82E-23	-4.350E-03	2.755E-03	0.114
rs9383843	6	150133954	A	C	-0.0332	0.0055	0.353	1.35E-09	1.863E-03	2.086E-03	0.372
rs10234636	7	40427617	T	C	0.0889	0.0081	0.111	4.43E-28	1.162E-03	3.252E-03	0.721
rs13235543	7	73013901	T	C	-0.0581	0.0080	0.127	3.06E-13	4.652E-03	2.968E-03	0.117
rs56067931	7	120481569	T	C	-0.0355	0.0065	0.200	4.83E-08	1.677E-03	2.495E-03	0.502
rs4733058	8	27281896	C	T	-0.0413	0.0070	0.165	3.06E-09	-6.625E-04	2.702E-03	0.806
rs4739105	8	64496159	T	C	0.0356	0.0064	0.211	2.85E-08	2.777E-03	2.489E-03	0.265
rs580845	9	14103618	C	A	-0.0299	0.0055	0.399	4.30E-08	-2.778E-03	2.070E-03	0.180
rs2383802	9	29350711	T	C	0.0372	0.0054	0.334	6.78E-12	2.785E-03	2.154E-03	0.196
rs7034179	9	71746838	T	C	0.0431	0.0052	0.429	1.60E-16	-3.962E-03	2.029E-03	0.051
rs17723637	9	109687403	G	A	0.0415	0.0072	0.150	8.63E-09	-3.155E-03	2.808E-03	0.261
rs3891689	9	119258583	C	T	0.0577	0.0061	0.234	2.28E-21	2.596E-03	2.363E-03	0.272
rs7916911	10	8722944	T	G	0.0398	0.0057	0.284	3.18E-12	2.820E-03	2.204E-03	0.201
rs10828247*	10	21822856	A	G	-0.0340	0.0059	0.653	7.51E-09	6.532E-03	2.100E-03	0.002
rs11187838	10	96038686	A	G	-0.0552	0.0052	0.439	3.70E-26	4.355E-03	2.011E-03	0.030
rs12260159	10	100702737	A	G	-0.0832	0.0103	0.075	7.33E-16	-9.531E-04	3.688E-03	0.796
rs12260436	10	104741114	C	A	0.0360	0.0058	0.257	7.29E-10	-1.933E-03	2.300E-03	0.401
rs869432	10	112502662	C	A	-0.0293	0.0053	0.416	3.54E-08	-2.450E-03	2.049E-03	0.232
rs2672592	10	124230750	T	G	0.0380	0.0054	0.363	1.22E-12	-1.670E-04	2.088E-03	0.936

rs11248546	10	125242283	T	C	-0.0368	0.0052	0.436	1.59E-12	2.162E-03	2.006E-03	0.281
rs4880425	10	134587640	A	G	0.0386	0.0061	0.510	2.73E-10	-3.446E-04	1.994E-03	0.863
rs4391795	11	3249552	T	C	0.0440	0.0060	0.419	1.54E-13	-9.372E-04	2.022E-03	0.643
rs4909945	11	10673739	T	C	-0.0568	0.0055	0.322	1.39E-24	-2.717E-04	2.150E-03	0.899
rs1003194	11	15126085	A	G	0.0343	0.0054	0.383	2.43E-10	1.105E-03	2.091E-03	0.597
rs11031122	11	30547438	C	T	0.0366	0.0059	0.243	6.91E-10	9.797E-04	2.317E-03	0.672
rs7932866	11	46548094	G	A	-0.0431	0.0072	0.164	2.38E-09	-5.143E-03	2.660E-03	0.053
rs566673*	11	66401373	G	T	0.0302	0.0053	0.462	9.07E-09	-5.770E-03	2.010E-03	0.004
rs11225163	11	102070843	T	C	0.0394	0.0054	0.347	2.63E-13	2.378E-03	2.105E-03	0.259
rs10894756*	11	133745852	A	G	-0.0295	0.0053	0.427	2.83E-08	3.840E-03	2.023E-03	0.058
rs2160875	12	4527322	C	T	0.0649	0.0052	0.477	2.72E-36	9.321E-04	2.016E-03	0.644
rs1458170	12	41901277	T	C	-0.0416	0.0071	0.156	5.75E-09	-1.466E-03	2.739E-03	0.592
rs11172113	12	57527283	C	T	-0.1068	0.0053	0.415	1.38E-90	-6.032E-03	2.023E-03	0.003
rs11105376	12	90088768	A	G	-0.0411	0.0071	0.168	7.93E-09	3.397E-03	2.667E-03	0.203
rs10777902	12	98498223	A	C	0.0331	0.0051	0.498	1.25E-10	1.916E-03	1.998E-03	0.337
rs1271309	12	124820705	A	G	-0.0403	0.0073	0.164	3.74E-08	-5.927E-04	2.718E-03	0.827
rs7335684*	13	47193696	G	A	0.0342	0.0060	0.247	1.05E-08	-7.756E-03	2.317E-03	0.001
rs7996252*	13	78876537	C	T	-0.0289	0.0053	0.404	4.11E-08	3.255E-03	2.030E-03	0.109
rs2000660	13	110788441	A	G	0.0496	0.0091	0.092	4.95E-08	-3.778E-03	3.529E-03	0.284
rs1245463	14	27661650	A	G	0.0396	0.0053	0.388	5.72E-14	5.427E-03	2.047E-03	0.008
rs1542668	14	42548912	A	G	-0.0307	0.0055	0.329	2.53E-08	-2.349E-03	2.125E-03	0.269
rs28756401	14	58761912	A	G	-0.0335	0.0058	0.285	6.40E-09	1.594E-03	2.206E-03	0.470
rs55707505*	14	75362552	C	T	-0.0307	0.0055	0.325	2.48E-08	3.922E-03	2.127E-03	0.065
rs11624776	14	93595591	C	A	-0.0498	0.0056	0.316	9.75E-19	1.403E-03	2.133E-03	0.511
rs28929474	14	94844947	T	C	0.1110	0.0186	0.019	2.54E-09	-6.042E-03	7.133E-03	0.397
rs12708529	15	81022364	G	A	-0.0358	0.0058	0.270	8.11E-10	9.476E-04	2.240E-03	0.672
rs12598836*	16	4534482	G	A	0.0378	0.0059	0.304	2.21E-10	-1.004E-02	2.186E-03	4.36E-06
rs34624768	16	75331572	G	A	0.0385	0.0052	0.404	1.71E-13	-2.801E-03	2.034E-03	0.168
rs8052831	16	87578039	G	A	0.0426	0.0055	0.344	8.25E-15	1.231E-03	2.109E-03	0.560
rs9894634*	17	1967501	C	T	0.0339	0.0052	0.403	9.64E-11	-5.891E-03	2.036E-03	0.004
rs34914463*	17	7366619	C	T	-0.0492	0.0082	0.129	2.41E-09	8.562E-03	2.943E-03	0.004
rs78378222	17	7571752	G	T	0.1248	0.0229	0.014	4.93E-08	1.313E-02	9.414E-03	0.163
rs2555111*	17	46648899	C	T	0.0287	0.0051	0.493	2.34E-08	-3.469E-03	2.004E-03	0.084
rs2119930	17	47514039	G	T	0.0409	0.0052	0.405	6.69E-15	-2.765E-04	2.075E-03	0.894
rs12452590	17	60720058	G	T	0.0375	0.0059	0.375	2.03E-10	-3.204E-03	2.119E-03	0.131
rs8077768	17	78256432	C	T	0.0397	0.0056	0.477	9.32E-13	-1.091E-03	2.000E-03	0.585
rs7504540	18	20201550	T	C	0.0364	0.0054	0.501	1.19E-11	4.429E-03	2.007E-03	0.027
rs1019990	18	44866736	T	C	-0.0387	0.0057	0.296	1.00E-11	1.763E-03	2.187E-03	0.420
rs8087942	18	55192245	G	A	-0.0392	0.0055	0.333	9.71E-13	-7.412E-04	2.120E-03	0.727
rs10405121	19	13339128	A	G	-0.0332	0.0053	0.452	4.74E-10	4.039E-03	2.022E-03	0.046
rs74182632*	19	19406126	A	G	0.0638	0.0112	0.055	1.43E-08	-1.052E-02	4.420E-03	0.017
rs11668109	19	41863777	A	C	-0.0374	0.0057	0.314	4.59E-11	4.367E-04	2.189E-03	0.842
rs616060	20	10547805	A	G	-0.0333	0.0053	0.371	4.04E-10	4.759E-04	2.065E-03	0.818
rs6046147	20	19479796	T	C	0.0645	0.0058	0.255	2.47E-28	-7.993E-04	2.285E-03	0.727
rs6057599	20	31168439	T	C	0.0413	0.0055	0.335	8.73E-14	-5.070E-03	2.132E-03	0.017
rs3092262*	20	45580290	G	A	0.0299	0.0052	0.463	1.08E-08	-4.915E-03	2.050E-03	0.017
rs910187	20	45841052	A	G	-0.0349	0.0054	0.372	1.14E-10	4.261E-03	2.060E-03	0.039
rs28451064	21	35593827	A	G	-0.0633	0.0080	0.131	3.52E-15	-6.951E-03	3.070E-03	0.024
rs764508	21	36935896	C	T	0.0315	0.0053	0.370	3.28E-09	-2.161E-04	2.078E-03	0.917
rs625686	22	20142932	C	T	0.0341	0.0059	0.306	8.26E-09	-6.695E-05	2.212E-03	0.976

* SNPs excluded from Steiger-filtered analysis.

Abbreviations: EA, effect allele; eaf, effect allele frequency; OA other allele; SNP, single nucleotide polymorphism.

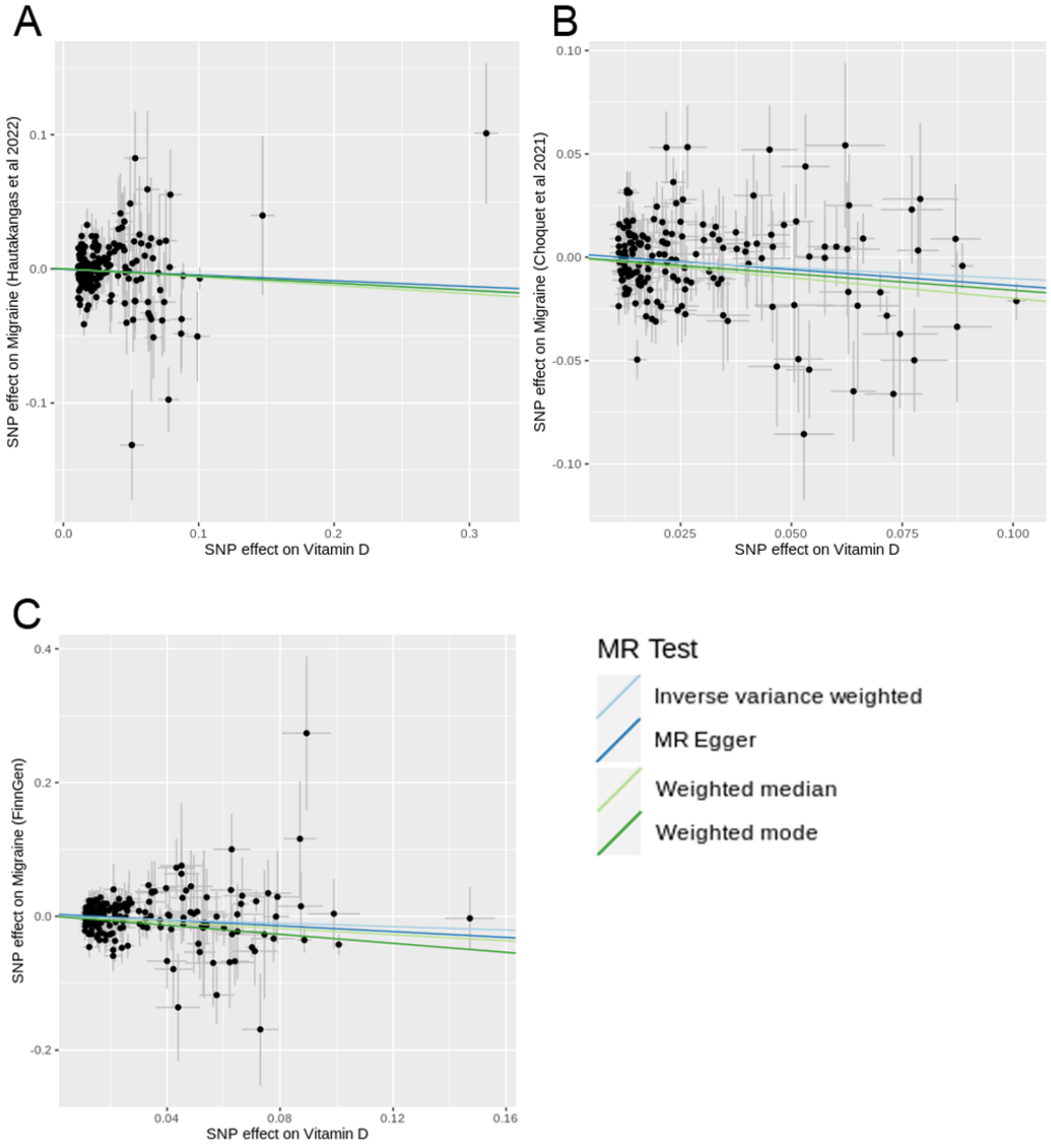

Supplementary Figure 1 Scatter plots of SNP-25OHD and SNP-migraine associations

A: migraine data from Hautakangas et al. 2022; **B**: migraine data from Choquet et al. 2021; **C**: migraine data from FinnGen (Release 6).

Abbreviations: 25OHD, 25 hydroxyvitamin D; MR, Mendelian randomization; SNP, single nucleotide polymorphism.

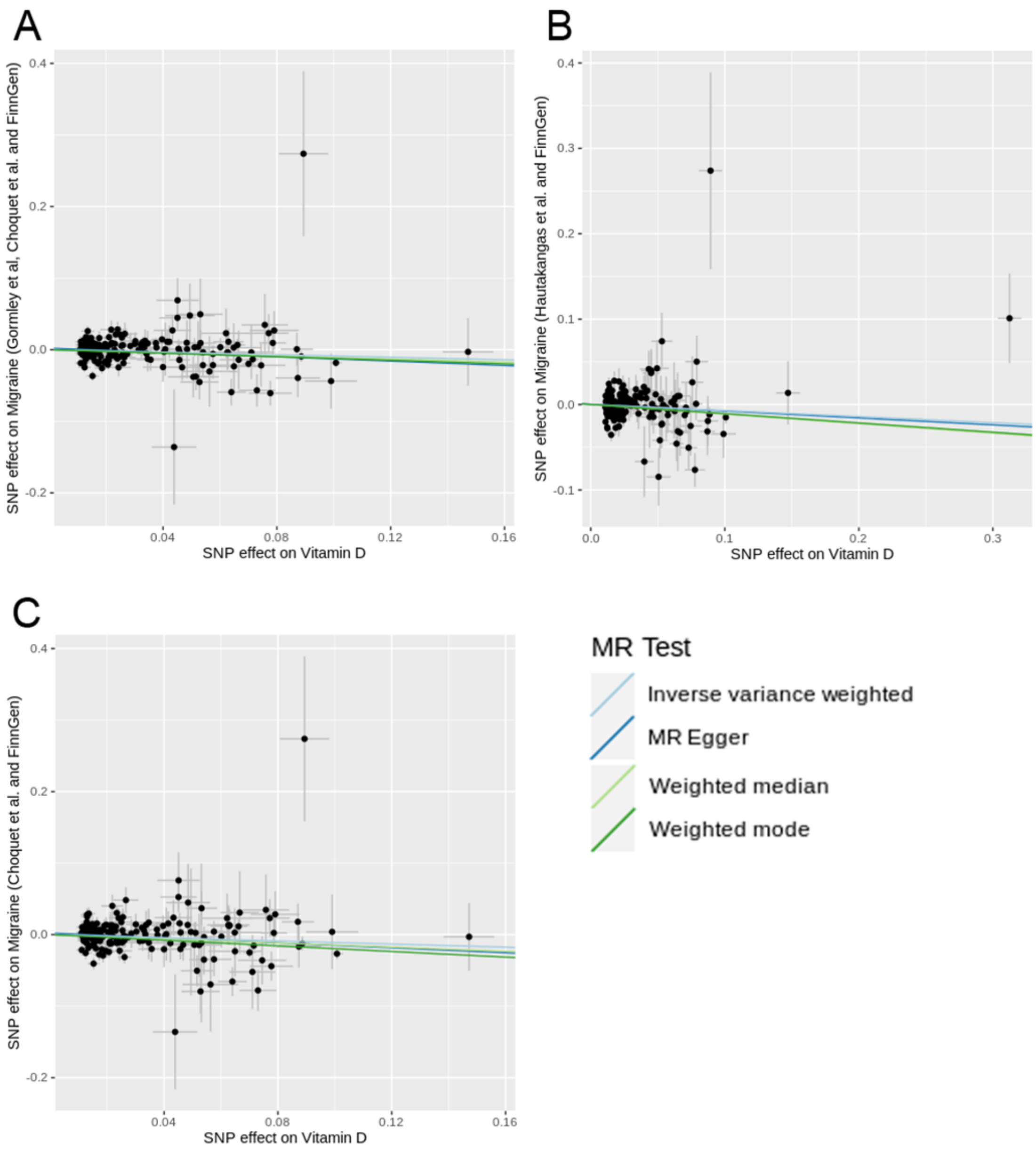

Supplementary Figure 2 Scatter plots using pooled migraine datasets

A: pooled data from Gormley et al. 2016, FinnGen (Release 6) and Choquet et al. 2021; **B**: pooled data from Hautakangas et al. 2022 and FinnGen (Release 6); **C**: pooled data from Choquet et al. 2021 and FinnGen (Release 6).

Abbreviations: MR, Mendelian randomization; SNP, single nucleotide polymorphism.

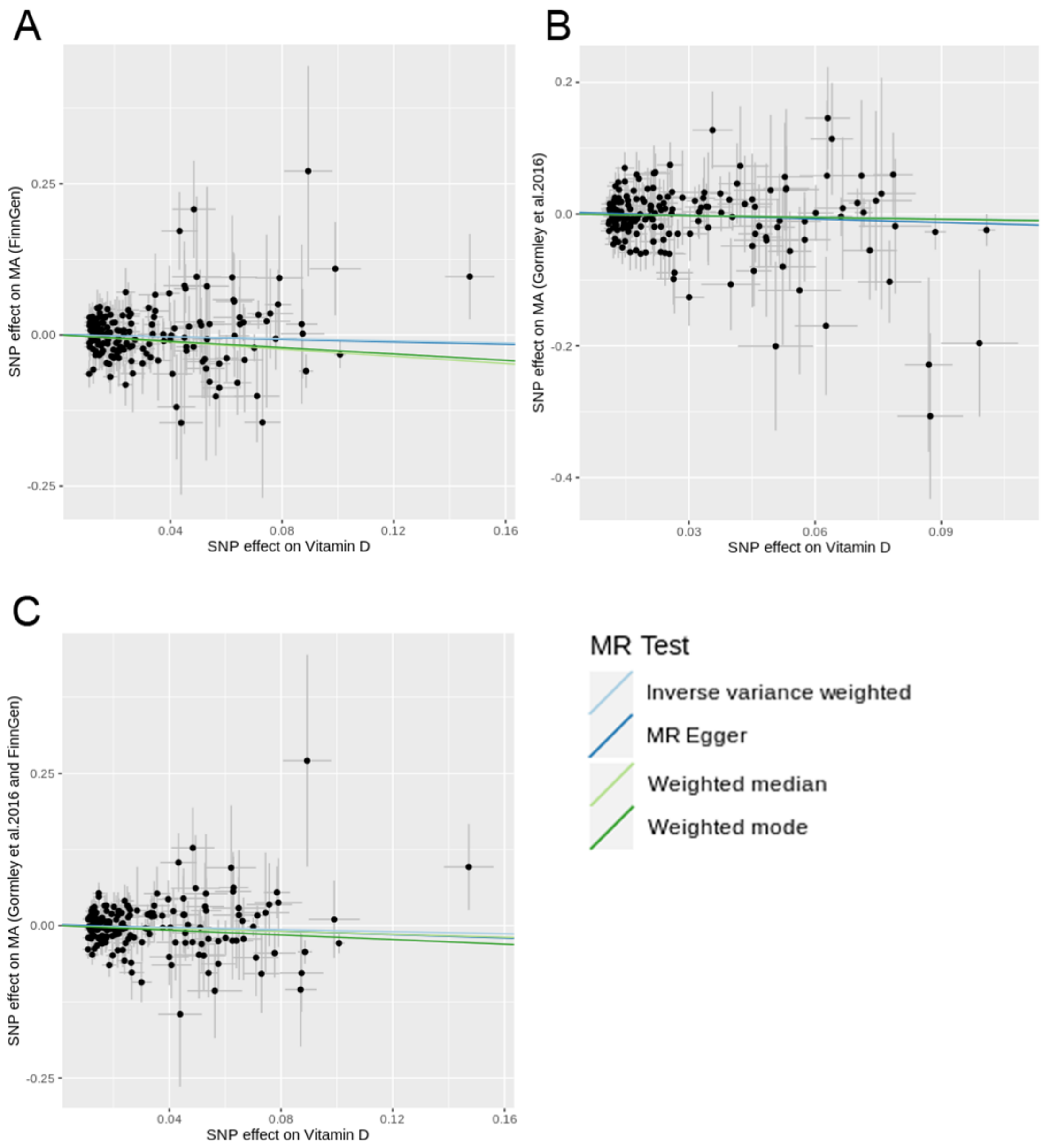

Supplementary Figure 3 Scatter plots of SNP-25OHD and SNP-MA associations

A: MA from FinnGen (Release 6); **B**: MA from Gormley et al. 2016; **C**: pooled MA of FinnGen (Release 6) and Gormley et al.

Abbreviations: MA, migraine with aura; MR, Mendelian randomization; SNP, single nucleotide polymorphism.

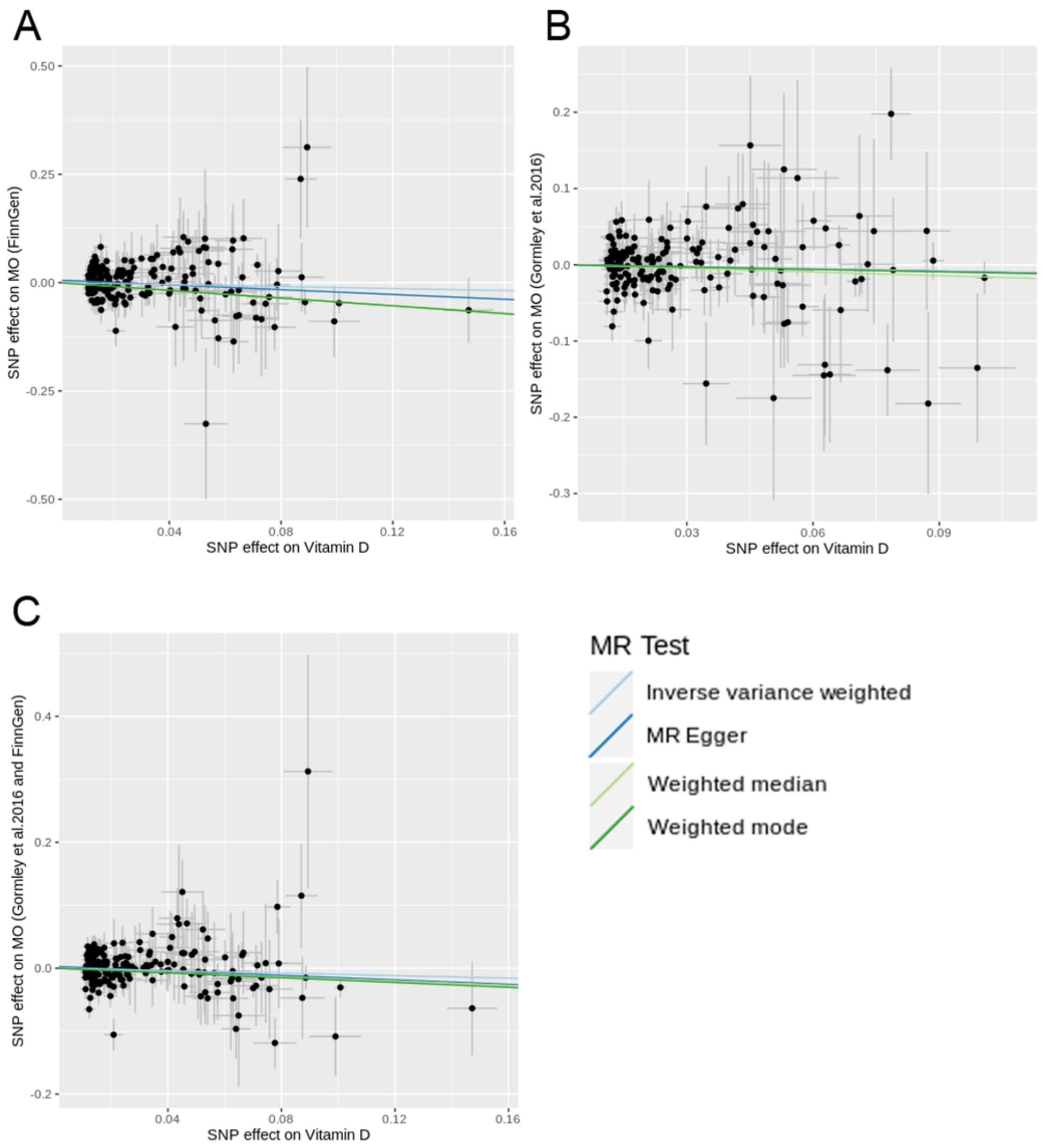

Supplementary Figure 4 Scatter plots of SNP-25OHD and SNP-MO associations

A: MO from FinnGen (Release 6); **B**: MO from Gormley et al. 2016; **C**: pooled MO of FinnGen (Release 6) and Gormley et al.

Abbreviations: MO, migraine without aura; MR, Mendelian randomization; SNP, single nucleotide polymorphism.

Outcome dataset and MR method	SNP, n	OR (95% CI)	p value
**Pooled MA (10,698 cases and 353,728 controls)**			
Inverse variance weighted	6	0.779(0.562-1.080)	0.134
MR Egger	6	0.730(0.404-1.319)	0.356
Weighted median	6	0.766(0.534-1.100)	0.149
Weighted mode	6	0.748(0.515-1.086)	0.188
**Pooled MO (12,272 cases and 348,467 controls)**			
Inverse variance weighted	6	0.885(0.649-1.207)	0.441
MR Egger	6	1.229(0.703-2.150)	0.510
Weighted median	6	0.913(0.637-1.308)	0.619
Weighted mode	6	1.032(0.709-1.500)	0.877

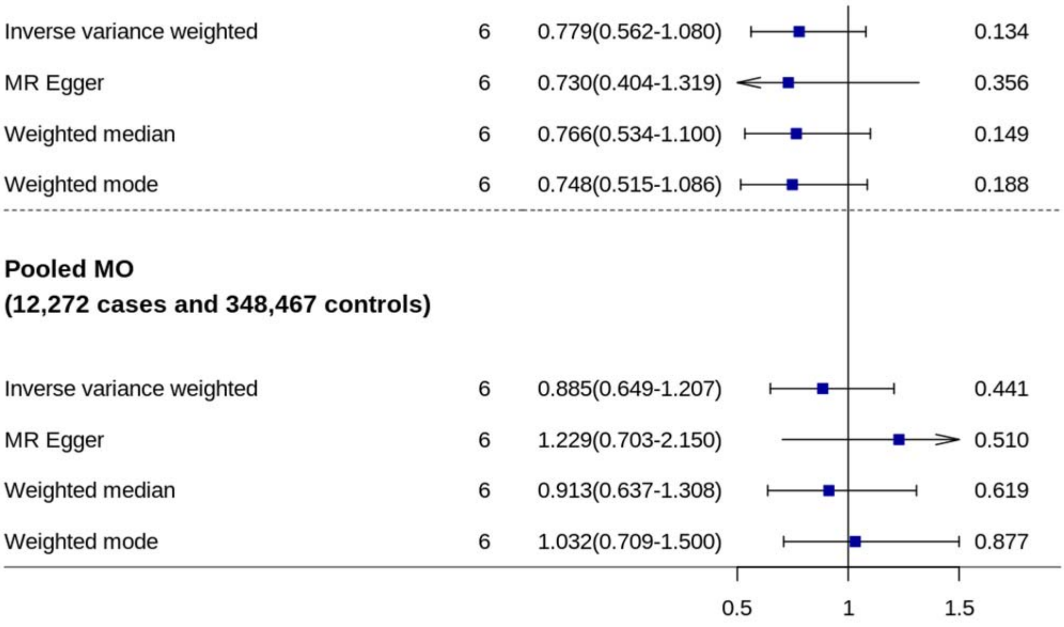

Supplementary Figure 5 Replication analyses of 25OHD on risk of migraine subtypes

No outlier was detected by MR-Pleiotropy Residual Sum and Outlier.

Abbreviations: CI, confidence interval; MA, migraine with aura; MO, migraine without aura; MR, Mendelian randomization; OR, odds ratio; SNP, single nucleotide polymorphism.

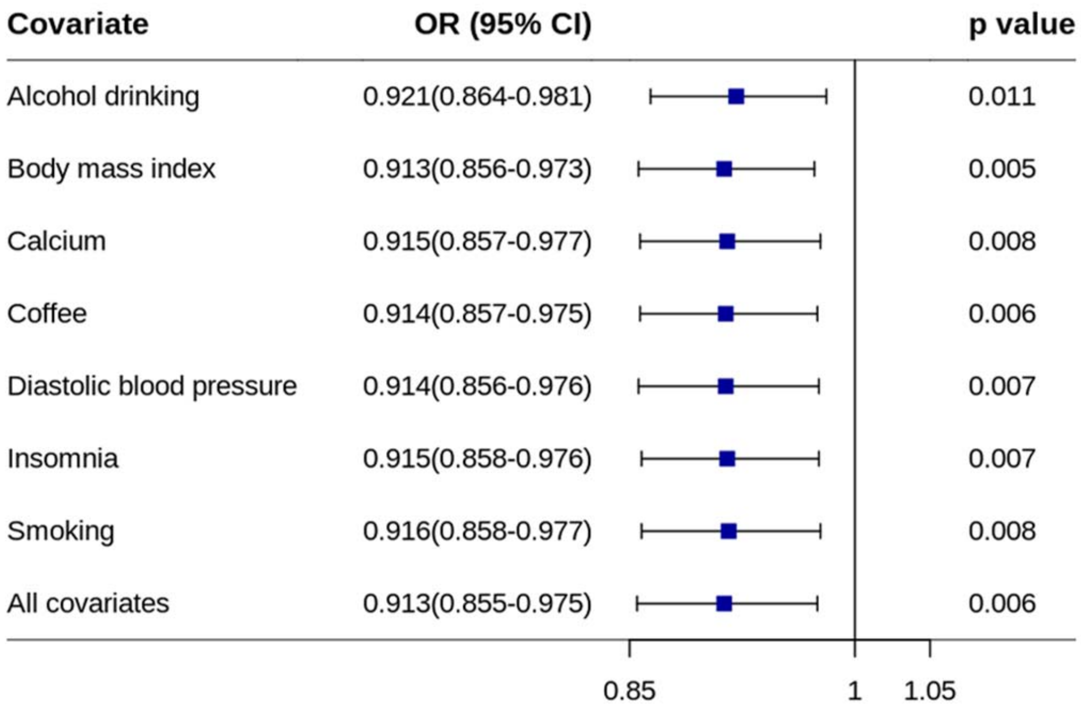

Supplementary Figure 6 Multivariable Mendelian randomization analyses
Abbreviations: CI, confidence interval; OR, odds ratio.

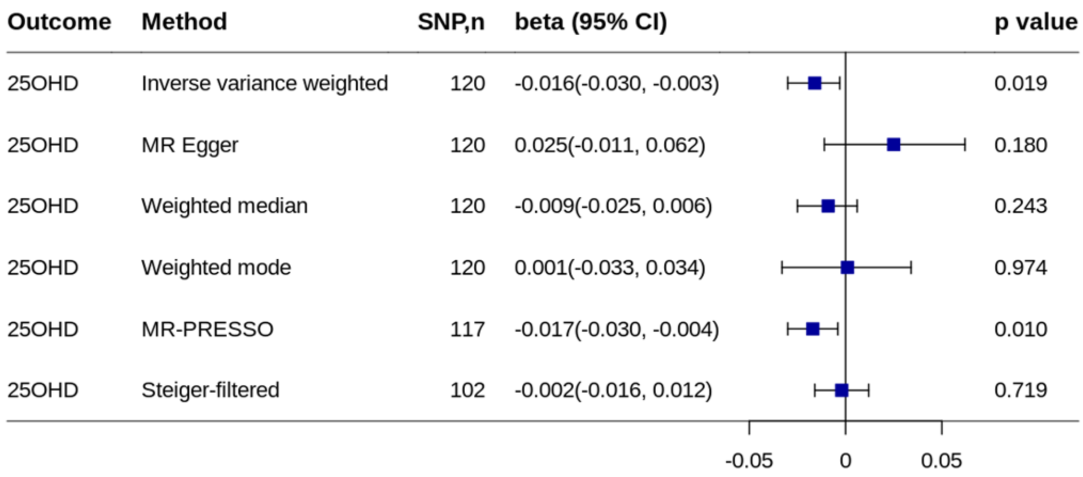

Supplementary Figure 7 Reverse Mendelian randomization analysis

Abbreviations: 25OHD, 25-hydroxyvitamin D; CI, confidence interval; MR, Mendelian randomization; SNP, single nucleotide polymorphism.
